# An Atomistic View of Amyloidogenic Self-assembly: Structure and Dynamics of Heterogeneous Conformational States in the Pre-nucleation Phase

**DOI:** 10.1038/srep33156

**Published:** 2016-09-12

**Authors:** Dirk Matthes, Vytautas Gapsys, Julian T. Brennecke, Bert L. de Groot

**Affiliations:** 1 Computational Biomolecular Dynamics Group, Max Planck Institute for Biophysical Chemistry, Göttingen, Germany

## Abstract

The formation of well-defined filamentous amyloid structures involves a polydisperse collection of oligomeric states for which relatively little is known in terms of structural organization. Here we use extensive, unbiased explicit solvent molecular dynamics (MD) simulations to investigate the structural and dynamical features of oligomeric aggregates formed by a number of highly amyloidogenic peptides at atomistic resolution on the *μ*s time scale. A consensus approach has been adopted to analyse the simulations in multiple force fields, yielding an in-depth characterization of pre-fibrillar oligomers and their global and local structure properties. A collision cross section analysis revealed structurally heterogeneous aggregate ensembles for the individual oligomeric states that lack a single defined quaternary structure during the pre-nucleation phase. To gain insight into the conformational space sampled in early aggregates, we probed their substructure and found emerging *β*-sheet subunit layers and a multitude of ordered intermolecular *β*-structure motifs with growing aggregate size. Among those, anti-parallel out-of-register *β*-strands compatible with toxic *β*-barrel oligomers were particularly prevalent already in smaller aggregates and formed prior to ordered fibrillar structure elements. Notably, also distinct fibril-like conformations emerged in the oligomeric state and underscore the notion that pre-nucleated oligomers serve as a critical intermediate step on-pathway to fibrils.

Amyloid fibril formation has been found to be a nucleation-dependent reaction for which the emergence of oligomeric entities is an inevitable and, presumably, critical intermediate step in the filamentous growth process^1^,^2^,^3^,^4^,^5^,^6^,^7^. Therefore, mapping out the molecular details of the early stages of the amyloidogenic self-assembly process is of vital importance to tackle a variety of fundamental challenges posed in contemporary physico-chemical and bio-medical research^4^,^5^. A particular question is revolving around the causal relation of impaired cellular viability and the presence of small and soluble proteinaceous aggregates as documented for several neurodegenerative and metabolic disorders^8^,^9^,^10^. The elucidation of highly ordered structures formed in the terminal stages of the aggregation cascade for numerous peptides and proteins, both disease related and biologically functional^11^,^12^,^13^,^14^,^15^,^16^, has deemed the cross-*β* conformation as the imperative packing motif for the polypeptide backbone in the amyloid state^4^,^16^,^17^,^18^,^19^. Short sequence segments from amyloidogenic proteins are established *in vitro* model systems for fibre formation and have been shown to reproduce all fundamental properties of fibrillar aggregates obtained from proteins with the full-length amino-acid sequence^17^,^20^,^21^. X-ray crystallographic studies on amyloid-like hexapeptides have allowed atomistic structure determination of the amyloid state for a wide range of sequences with different amino acid compositions. As a refined model of the fibrillar spine at atomic resolution, intermolecular *β*-sheets commonly stacked via self-complementary side-chain interfaces - so called steric zippers - were revealed^20^,^22^. Clearly, these short peptides cannot encompass the complete intricacy that might be expected for the oligomer and fibril formation process, or even the aggregation events *in vivo*, of the full-length polypeptides containing the very same segments. However, the small peptides and their local structure properties have been found to be essential to the aggregation process in various instances, e.g. by showing the ability to seed the fibrillization of their parent proteins^20^,^21^,^23^. The relevance to the aggregation pathway becomes furthermore apparent as fibrous crystals of short peptide systems are suitable platforms to design strategies that prevent cytotoxic aggregate formation^1^,^24^.

Metastable, non-fibrillar structures were described for several preparations of amyloid-forming peptides and proteins^1^,^6^,^9^,^25^,^26^,^27^,^28^,^29^. It has, however, not been straightforward to translate the extensive structural knowledge gained for the stable, fibrillar and crystalline states to the soluble oligomers prevalent in the pre-nucleation phase^1^. In a number of recent studies, designed macrocyclic peptide model systems, which are chemically constrained in their sampling of fibrillar aggregate conformations, have been explored to investigate the otherwise transient and highly aggregation-prone oligomeric states^30^,^31^,^32^,^33^. The structures of such *β*-sheet amyloid mimics in the solid- and solution-state suggest that maximization of both hydrophobic contacts and hydrogen bonding are key aspects governing the stabilization of low-molecular-weight oligomers^25^,^30^,^31^,^32^. Furthermore, structural studies of model peptide oligomers *in situ* using ion-mobility mass spectrometry (IM-MS) implicated conformational rearrangements from initially unstructured and coalesced chains toward ordered *β*-sheets resembling the generic steric zipper motif 34,35. Based on the evidence presented, tightly laminated *β*-sheets with dry interior may be a common feature of oligomeric aggregates and might thus help to rationalize the assembly into topologies with crossing *β*-sheet and *β*-barrel structures^31^,^36^,^37^.

Nevertheless, correlating physico-chemical properties and atomistic structural information for a polymorphic and dynamical interchanging oligomer structure ensemble has been an intricate task in experiments^1^,^10^. It has been shown that low charge states and an increased hydrophobicity introduced by mutation promote the aggregation rates of unfolded or intrinsically unstructured polypeptides^38^,^39^. At the same time, the propensity of short peptide segments to aggregate into well defined amyloid-like steric zipper structures has been found to be highly sequence dependent, albeit relatively insensitive to the amino acid composition^35^,^40^. Moreover, several studies convincingly demonstrated that the ability to oligomerize is an insufficient condition to judge whether a given sequence eventually forms fibrillar aggregates^35^,^39^. A high-resolution structural description of the oligomeric state ensemble and accessible conformational space is therefore necessary to understand molecular phenomena determining the rate limiting steps in the primary nucleation pathway^3^,^41^. To this end, atomistic simulations have been applied to unravel mechanistic events beyond averaged pictures of molecular processes or static views from single crystal structures^41^,^42^,^43^,^44^,^45^,^46^,^47^,^48^,^49^,^50^,^51^,^52^,^53^. We refer to refs 54 and 55 for a comprehensive review of important and recent literature on this topic. Briefly, several contributions reporting on simulations of small amyloidogenic peptide aggregation give strong evidence that oligomeric aggregates form via aggregation states with collapsed and partially ordered chains, subsequently leading to *β*-sheet rich structures^45^,^46^,^50^,^53^,^54^,^55^,^56^. The observed relative free energy differences in the individual states indicate that anti-parallel *β*-strand conformations are initially favoured, up until the cooperative formation of an increasing fraction of parallel *β*-sheet structure with growing aggregate size^44^,^46^,^48^,^50^,^51^,^52^,^57^. The heterogeneous oligomeric aggregates are furthermore commonly reported to adopt a diverse set of transient structural arrangements^42^,^45^,^53^,^56^ reminiscent of layered *β*-sheet^41^,^43^,^45^,^48^,^53^,^58^ and *β*-barrel conformations^43^,^47^,^49^.

Simulating the peptide self-assembly process with an atomistic representation of molecular dynamics is demanding and has been subject to incremental methodological advancements over the years^54^,^55^. Approximative approaches have been widely used in the past, such as simplified representations of the protein force field^47^,^48^,^49^,^51^,^53^ and implicit solvent models^49^,^51^,^52^,^53^. Whereas recent all-atom simulations with general purpose force fields typically involve very high peptide concentrations in the range of 100 mM^46^,^50^,^55^,^58^,^59^ and often make use of temperature enhanced^41^,^43^,^46^,^52^ or biased sampling^50^,^59^ techniques, that result in the loss of dynamical information for the studied systems.

In this contribution, we utilized unbiased Molecular Dynamics (MD) simulations on the *μ*s time-scale to capture the general principles underlying the molecular self-assembly pathways of short peptides. More specifically, we examined if common secondary, tertiary and quaternary structural characteristics emerge in the pre-nucleation phase of low-molecular-weight amyloid oligomers. Furthermore, given the recently available high resolution structure data from solid-state experiments, we were interested in understanding the role of ordered inter-molecular substructure motifs in the oligomerization process. We studied in total six peptides of six to seven residues using an explicit representation of the solvent molecules and at least three molecular mechanics force fields per simulation system. Four of these peptides have been proven experimentally to be highly amyloidogenic and their microcrystal structures have been determined: the segments ^16^KLVFFAE^22^ 22 and ^30^AIIGLM^35^ 22 from the amyloid-*β* peptide, the ^306^VQIVYK^311^ 20 segment of the htau40 protein, and the ^8^NNQQNY^13^ segment^20^ of the yeast prion-protein sup35. To test sequence specific effects, we additionally studied a mutated version of the yeast prion sequence, VIQVVY^35^. The non-fibrillizing ^207^GSRSRT^212^ 23 segment of htau40 was included as a control.

In the following sections we first quantify important aspects of the spontaneous oligomerization process for the six selected sequences. Next, we gauge the dynamical and structural aggregation process characteristics on the level of individual oligomeric states formed along the oligomerization pathway. We assess the generality of findings with respect to available experimental data from X-ray crystallography and solid-state NMR and discuss the implications for amyloidogenic peptide aggregation.

## Results

### Amyloidogenic peptides spontaneously populate higher-order oligomeric aggregates, but the extent varies with force field

**Figure 1:**
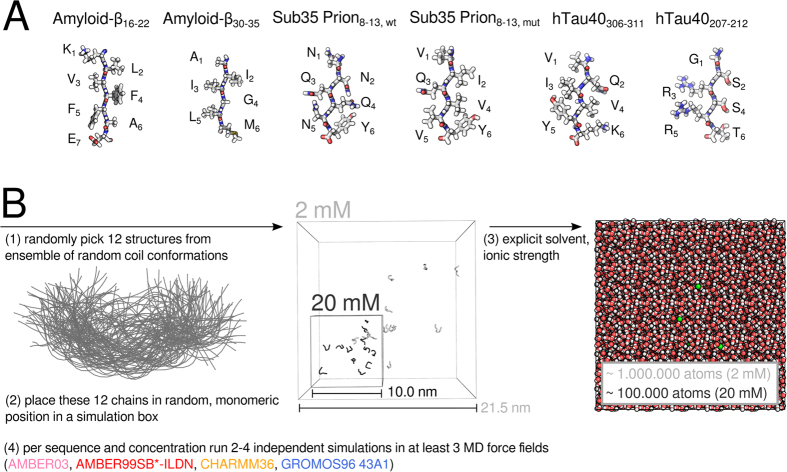
Simulation setup. (**A**) Studied peptide sequences visualized in stick representation using PyMol^106^. (**B**) General protocol to set up simulations of spontaneous oligomerization at two different peptide concentrations, with representative snapshots of simulation boxes.

The six studied peptide sequences were probed in their propensity to oligomerize into multimeric peptide aggregates by MD simulations using multiple atomistic force fields and two peptide concentrations (2 mM and 20 mM). For each studied combination of sequence, force field and peptide concentration, we carried out at least two independent simulations. The total number of obtained trajectories and respective simulation lengths is given in Table 1 and Supplementary Table 1. An overview of the simulation systems and setup is shown in Fig. 1.

**Figure 2:**
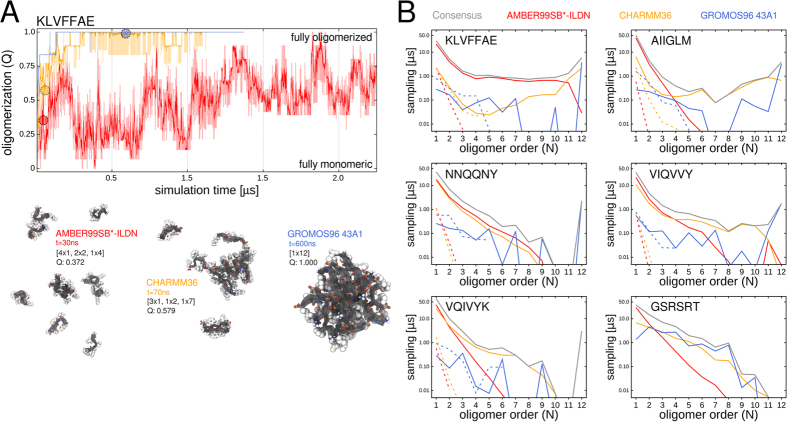
Spontaneous self-assembly into higher-order oligomers. (**A**) Time resolved association to higher-order oligomers is shown for an exemplary set of KLVFFAE simulations. Representative structures are shown in ribbon and sphere representation using VMD^107^ and Tachyon^108^ (solvent molecules and ions omitted). (**B**) The obtained consensus sampling statistics of the individual aggregation states in three different MD force fields is shown for each of the six studied peptide sequences in simulations of 2 mM and 20 mM peptide concentration (dashed and continuous lines). Red, orange and blue coloured traces denote individual simulations carried out with AMBER99SB*-ILDN, CHARMM36 and GROMOS96 force fields, respectively. This colour scheme is used throughout the paper.

For a preliminary characterization of the oligomerization process, we monitored the extent of oligomerization (*Q*) i.e. the formation of higher-order oligomers during the simulations (see Methods). Figure 2A shows the time evolution for a set of KLVFFAE peptide oligomerization trajectories simulated with three different force fields at 20 mM concentration (AMBER99SB*-ILDN, CHARMM36, GROMOS96 43A1). Starting always from initially monomeric random coil peptides, the simulations spontaneously sampled various association states and aggregate configurations up to the eventual formation of a fully oligomerized, dodecameric state. In total 75 *μ*s of simulation time were collected and allowed to obtain a consensus sampling of individual aggregation states in the *μ*s regime (Fig. 2B).

**Figure 3:**
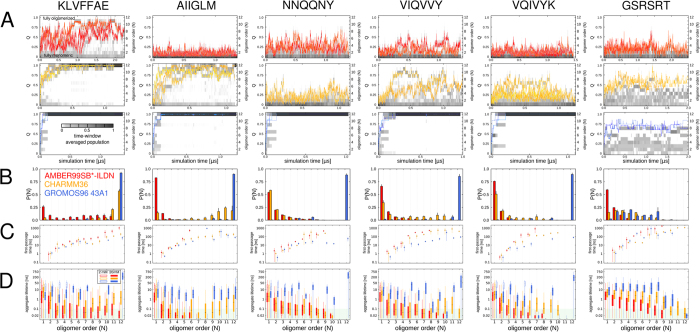
Extent of oligomerization and association dynamics vary with force field. (**A**) Oligomerization progress is shown for each of the six studied peptide sequences at 20 mM peptide concentration. Red, orange and blue colored traces denote individual simulations carried out with AMBER99SB*-ILDN, CHARMM36 and GROMOS96 force fields, respectively. Average populations of individual oligomerization states are shown as a function of simulation time averaged over all trajectories. Dark-gray and gray colors indicate a high abundance of oligomer order *N*. Respective block averages over 10 ns are shown to improve clarity. (**B**) Bar histograms in each multi panel depict the normalized oligomer size distributions determined over all trajectories of a particular sequence and force field combination. (**C**) Average first-passage time for aggregates of size *N* on a logarithmic scale (20 mM). (**D**) Uninterrupted lifetimes are shown for aggregates of order *N* in box plots, enclosing 25% and 75% of the individual distributions in the lower panels (2 and 20 mM). The median lifetime is denoted by the horizontal black lines, the observed respective minimal and maximal lifetimes are indicated by the whiskers. The shaded area indicates the fraction of transient oligomer structures with lifetimes below 100 ps.

An analysis of oligomerization propensity was carried out for every simulation system and showed that all amyloidogenic sequences readily self-assembled at millimolar peptide concentration through the stable formation of higher oligomeric aggregates at least for two MD force fields (Fig. 3A). Normalized oligomer size distributions were calculated over all independent trajectories for a given simulation system (Fig. 3B) and show that the spontaneous sampling of higher-order oligomeric states with *N* > 4 was more pronounced for peptides with multiple hydrophobic residues (KLVFFAE, AIIGLM and VIQVVY) in relative comparison to the more hydrophilic peptide sequences (NNQQNY and GSRSRT, Fig. 3B). The small standard deviations for each individual state in the size distribution indicate a consistent sampling pattern for each particular peptide sequence and force field combination in the simulated time scales for all simulation runs (Fig. 3B).

To trace and quantify the occurrence of productive oligomerization events, we determined the first-passage time *τ*_*N*_ for oligomeric structures of size *N* which persist for more than 100 ps in all trajectories (Fig. 3C). Smaller aggregates (up to the trimer and tetramer) indeed emerge quickly via a heterogeneous assembly pathway as observed in a similar manner for all tested force fields. Oligomer sizes larger than the tetramer formed in a consecutive, stepwise manner as can also be seen from the time series of averaged oligomer size populations (Fig. 3A). The corresponding observed first-passage times for higher-order oligomeric states *τ*_*N* > 4_ ranged from 10 ns to hundreds of ns and scattered among the independent trajectories of the same simulation system by the same order of magnitude (Fig. 3C). We found a faster non-transient oligomer formation on the sub-*μ*s time scale in the GROMOS96 compared to the AMBER99SB*-ILDN and CHARMM36 force field among the multiple independent simulation runs per sequence.

For all oligomer sizes *N* sampled in the simulations at 2 and 20 mM peptide concentration, we also calculated the uninterrupted aggregate lifetimes and found that the majority of the observed oligomeric states for any sequence was rather short-lived (Fig. 3D). With the exception of the fully oligomerized state, we found comparable median and also maximally observed lifetimes in the range of nanoseconds to tens of nanoseconds consistently across all sampled aggregate states per peptide sequence and force field (Fig. 3D, black horizontal lines and upper whiskers). Aggregates with lifetimes of 100 ps or shorter were considered only marginally stable and therefore discarded from the subsequent analysis (Fig. 3D). The observed maximal lifetimes of the 12-mer, which were dictated only by the rate of dissociation, were found in the range of hundreds of ns in the GROMOS simulations. The smaller aggregates in the GROMOS trajectories are brief intermediates to the eventual formation of dodecameric assemblies and the lifetimes of these states were mainly determined by the high oligomerization rate, which effectively reduced the concentration of molecular entities with time (Fig. 3A,D). In contrast, monomeric peptides and smaller multimeric precursors were abundantly present also on long time scales in the AMBER and CHARMM simulations. This led to the frequent encounter of single or multiple peptide chains and therefore limited the lifetime of all the individual oligomeric states in addition to association and dissociation events. For simulations with lower initial peptide concentration (2 mM), our analysis shows an (expected) up shift in aggregate lifetime due to the reduced probability of diffusional encounter of individual molecules.

The present simulations therefore suggest a systematic difference in the absolute extent of oligomerization for the individual peptide systems in the simulations and primarily were found to be dependent on the employed MD force field. We identified a particularly low tendency to form higher-order oligomeric states for the AMBER99SB*-ILDN force field based on the observations made in the simulations of multiple sequences in the simulated time scales (Fig. 3A,B). We observed small to intermediate oligomer sizes as well as a significant amount of free peptide monomers over the whole simulation time course in AMBER99SB*-ILDN and CHARMM36 (Fig. 3A,B) for the aggregation-prone peptides. Persistent fluctuations in the extent of oligomerization *Q* on the *μ*s time scale illustrate the ongoing exchange among the oligomeric states of various sizes in the simulations with these force fields (Fig. 3A). In the GROMOS trajectories and in contrast to the simulations with CHARMM36 and AMBER99SB*-ILDN, the oligomeric aggregates grew rapidly and irreversibly in size with the exception of the non-amyloidogenic GSRSRT peptide (Fig. 3A). For the control sequence GSRSRT we performed on average the longest simulations among all studied peptides, where at least one trajectory for each force field ran up to 2 microseconds or beyond. We thereby could verify that this peptide indeed does not aggregate in orderly fashion on the microsecond timescale. By this we furthermore show and ensure that the combined force field approach used in the present study indeed discriminates between aggregation-prone and non-fibrillizing peptide sequences.

### Validation of consensus force field approach

To address the observed systematic differences in oligomerization propensity and to discern potential concerns about the force field choice and quality, we performed three sets of additional simulations summarized in Supplementary Table 1 and Supplementary Figures 1–3. First, as a reference to investigate the behavior of low-molecular-weight aggregates with elongated *β*-sheet structures in the various force fields (AMBER99SB*-ILDN, CHARMM36, GROMOS96 43A1), we carried out simulations of pre-assembled single or double layered *β*-sheet dodecamers based on crystal structure conformations^20^. In all force fields, the modeled *β*-sheet oligomers did not retain their ideal, planar solid-state configurations as shown by a RMSD analysis presented in Supplementary Fig. 1. The observed relaxation of the *β*-aggregates in an explicit solvent environment towards twisted and wrapped structures is concurrent with simulation studies on a variety of modeled filament sizes and packing topologies for several short peptide sequences^45^,^58^,^60^,^61^. Single *β*-sheet filaments were found to shear and sometimes rupture, whereas double layer *β*-sheets with steric zipper-like structure properties appear to be more stable within the simulated time scales (Supplementary Fig. 1). The results for the crystallographic reference states obtained with the different force fields are in good agreement with one another. The steric zipper structures of the KLVFFAE and NNQQNY peptides are particularly stable and rapidly disintegrate in the case of the AIIGLM sequence (Supplementary Fig. 1). For the hydrophobic AIIGLM peptides, which can only form hydrogen bonds through main-chain atom contacts, also the largest force field dependent difference in spontaneous aggregation behavior was found. We therefore verified as a second measure, whether spontaneously formed and fully oligomerized AIIGLM aggregate conformations from GROMOS trajectories would still be stable when simulated in the CHARMM36 and AMBER99SB*-ILDN force fields. However, this was not the case, as we found at least a partial disassembly of the aggregates already on the hundred ns time scale (Supplementary Fig. 2). Lastly, we included an additional set of simulations to test the spontaneous aggregation behavior of mainly the AIIGLM peptide with a few other standard protein force fields (Supplementary Table 1 and Supplementary Fig. 3) Most notably, all of the additionally tested MD force fields (AMBER03, OPLS-AA/L, CHARMM22*, GROMOS96 54A7) perform largely indistinguishable to GROMOS96 43A1 in terms of a high aggregation propensity for AIIGLM, showing a fast and irreversible oligomerization (Supplementary Fig. 3).

Interestingly, in contrast to the findings for AIIGLM, multiple simulations of the NNQQNY, VIQVVY and GSRSRT peptide were observed to have a very similar behavior in terms of oligomerization propensity for AMBER99SB*-ILDN and the AMBER03 force field, which differ in their charge sets^62^.

Along these lines, a recent study suggests that the coordination number of water molecules around the carboxylate and amino groups is too high in certain protein force fields, thus making desolvation and therefore also *β*-aggregation unfavorable^63^. The set of AMBER99SB RESP charges leads to an apparent overestimation of the hydration of both, the carboxylate and amino groups^63^. In accordance with these results, we found from several AMBER99SB*-ILDN test simulations that AIIGLM peptides with capped terminal residues showed a marked increase in association behavior over simulations of the AIIGLM peptide and even a hydrophobic mutant of this sequence with free termini (Supplementary Fig. 3). The non-polarizable CHARMM36 force field used in this study well reproduced the experimental reference for the solvation shell of the amino group. According to Goetz *et al*. this demonstrates that a fixed point-charge model can indeed describe aggregation adequately, although an overhydration of the carboxylate group was found as well^63^.

To summarize our findings: In comparison to the recent CHARMM36 and multiple AMBER force field variants, the simulations carried out with the GROMOS96 43A1 force field showed a preferred sampling of the collapsed, oligomeric state for aggregation-prone peptide sequences at high peptide concentrations. Other commonly used protein force fields such as OPLS-AA/L and CHARMM22* also showed a high association tendency in the case of the tested hydrophobic peptide sequence AIIGLM. The structural characteristics and stability of nucleated *β*-sheet structures, however, were less affected by the choice of force field.

As a pragmatic solution, we present a consensus approach, using multiple force fields throughout all the subsequent analyses and thereby ensuring that not a single conclusion rests on the results of one force field parameter set alone. Additionally, we simulated all amyloidogenic peptide systems at two peptide concentrations. In this way, we circumvented that systems with high oligomerization propensity would be strongly biased towards higher-order oligomers in their sampling statistics due to high concentration (Fig. 2B).

### Higher-order oligomers are composed of layered *β*-sheets

**Figure 4:**
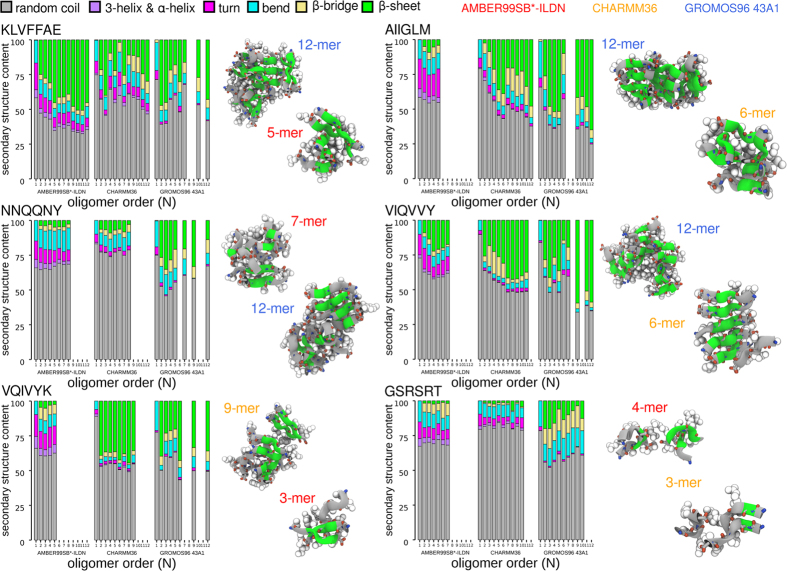
Secondary structure content of oligomeric states. Normalized histograms of secondary structure elements as defined by the DSSP algorithm within each oligomeric state of order *N*. Representative structures are shown in ribbon and sphere representation (*β*-structure colored green).

Next, we calculated the secondary structure content as ensemble average for each individual oligomeric state. For the amyloidogenic sequences we found a moderate to high average content of *β*-sheet and *β*-bridge secondary structure for all oligomeric aggregates starting from the peptide dimer accompanied by a decrease in the coil content (Fig. 4). The simulations carried out in the three different force fields agreed reasonably with one another in the trends observed for the different sequences and oligomerization states (Fig. 4).

Taking a closer look at the secondary structure on the level of individual aggregates reveals that the sizes of the *β*-sheets within the spontaneously assembled oligomers increased with the growing number of monomeric units present in a particular oligomeric state (Supplementary Fig. 4). For example, *β*-sheets in pentameric oligomers (*N* = 5) were found to be composed of up to five strands (Supplementary Fig. 4). A significant fraction of *β*-sheets in dodecameric oligomers (*N* = 12) extended up to six or even seven strands in length, especially for the aggregates of the more hydrophobic KLVFFAE, AIIGLM and VIQVVY peptides (Fig. 4 and Supplementary Fig. 4). For the remaining amyloidogenic sequences, NNQQNY and VQIVYK, the relative abundances of *β*-structured oligomers were smaller and varied depending on the individual aggregate sizes.

### Collision cross section analysis reveals heterogeneous ensemble of aggregates that lack a defined quaternary structure

To characterize the ensemble of oligomeric structures observed during the simulations, we computed collision cross sections (CCSs) of the individual aggregates sampled from the MD trajectories. CCSs report on the size and shape of soluble molecular assemblies^64^,^65^,^66^. CCSs have been measured experimentally for a range of non-covalently bound aggregate structures of small peptides during the first stages of the oligomerization and nucleation process^34^,^35^,^41^,^65^,^66^. The accurate determination of CCS from atomic coordinates, however, is a computationally challenging task^64^,^67^ and can become impractical to carry out for very large structural ensembles. Our simulations yielded a total number of 10^8^ aggregate structures with various sizes. We therefore chose to randomly select a set of 1000 structures per oligomer size for each sequence and force field combination and calculated the corresponding CCSs with the Trajectory method (see Methods). Subsequently, a Partial Least Squares (PLS) regression model was trained to compute CCS data for the full set of oligomeric structures based on several input variables that can be calculated at a lower computational cost (e. g. radius of gyration and hydrogen bond energies, see Methods). The correlation coefficients obtained by a cross-validation of the PLS models mostly ranged between 0.7 and 0.9 and assured that we were indeed able to reliably predict CCS values for the full structure ensembles (Supplementary Table 2).

**Figure 5:**
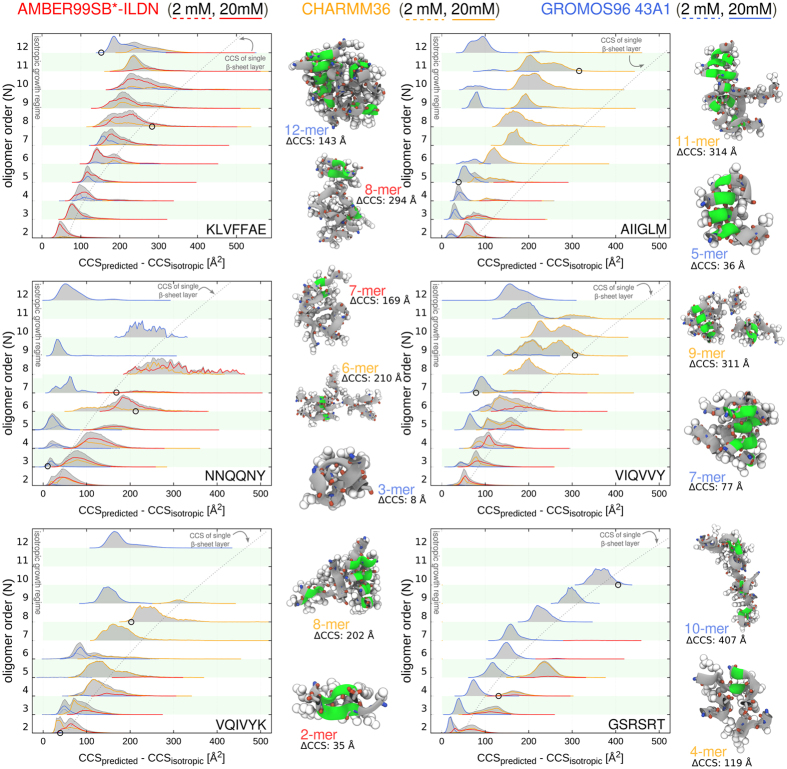
Collision cross section analysis of aggregate structure ensembles. Normalized histogram curves for the distributions of predicted and calculated collision cross sections (CCSs) are shown for each sampled oligomeric state of each peptide sequence. Red, orange and blue colors denote the structure ensembles obtained from AMBER99SB*-ILDN, CHARMM36 and GROMOS96 simulations, respectively. For comparison, simulations of 2 mM and 20 mM peptide concentration are presented in dashed and continuous lines. Gray shadings mark the consensus distributions obtained after pooling all simulations, respectively. For each oligomer size and sequence the CCSs are shown as the deviation from the theoretically expected CCSs of ideal, isotropic structures. Representative structures are shown in ribbon and sphere representation (*β*-structure colored green).

Figure 5 shows the size-dependent CCS distributions for each populated oligomeric state. It is important to note that the CCSs from the simulated aggregate ensemble snapshots were obtained nearly instantaneously after a short energy minimization in vacuum (see Methods). Thus, we resolved the structural features of oligomeric aggregates with solution-state properties and lifetimes, which are several orders of magnitude shorter compared to the millisecond experimental measurements^34^,^65^. To facilitate the interpretation of the data, all CCSs are shown as the deviation from the theoretically expected lower CCS limit of each oligomer size, namely ideal, isotropic structures that extend uniformly in all dimensions. The regime of isotropic oligomer growth was empirically estimated using the cross section of the monomer^34^ (see Methods). In addition, we highlighted the regions in the plot that correspond to the CCS of a *N*-stranded, single layered *β*-sheet structure to provide a theoretically estimated upper limit for the CCS of a fully ordered oligomer of size *N* (gray dashed lines in Fig. 5).

The CCS distributions shown in Fig. 5 are broad and suggest heterogeneous and multiple coexisting conformations for the individual oligomeric states of each peptide sequence. The aggregate cross sections from simulations carried out at 2 and 20 mM peptide concentration were found to be very similar in terms of distribution widths for each of the sampled oligomer sizes. Although the relative populations of aggregate structures as judged by the CCS were not identical for these two concentrations, also no significant shift could be observed (Fig. 5). In addition, irrespective of the peptide sequence, the simulated structure ensembles were overall found to have at least moderate deviations from ideal isotropic aggregates. Fully isotropic aggregates were not observed for any of the studied peptide aggregate sizes with the force field variants used in the current study.

The CCS distributions indicate that the majority of the aggregate conformations for all amyloidogenic peptide sequences fall into the anisotropic structure regime whose cross sections are compatible with layered *β*-sheet interfaces (Fig. 5). We found that the sub-ensemble of *β*-sheet rich oligomers was indeed predominantly densely packed instead of sampling elongated and highly ordered *β*-sheet filaments, which was also in line with the secondary structure analysis (Figs 4 and 5 and Supplementary Fig. 6). The noticeable widening of the CCS distributions for each of the higher-order oligomeric states was due to a high plasticity in the quaternary structure composed of multiple smaller *β*-sheet arrays (Fig. 5).

Oligomeric aggregates from the far right portion of the CCS distributions correspond to structures that are compatible with, or even exceed the theoretical cross sections of ordered *β*-sheet filaments. An inter-peptide contact analysis (Supplementary Fig. 6) reveals that the oligomers with the largest deviations from isotropic CCS values in each of the oligomeric states *N* have a low packing density and few inter-molecular contacts. On the structural level, the observed large cross sections arise from imperfectly formed *β*-strand arrangements or rather loosely and irregularly tethered peptide molecules as can be seen from representative simulation snapshots (Supplementary Fig. 6). We also observed an overall shorter lifetime for these seemingly transient aggregates compared to the oligomer structures with smaller cross sections and much more compact peptide arrangements, resulting in a tight packing of main- and side-chain groups within the aggregates (Fig. 5 and Supplementary Fig. 6).

The CCS analysis clearly shows that in all cases, the structural ensembles of oligomers obtained from GROMOS simulations were found with predominantly compact conformations in comparison to the ones obtained with AMBER and CHARMM force fields. In contrast, aggregates with a particularly high shape anisotropy and fewer inter-molecular contacts were prevalently observed in simulations with the CHARMM and AMBER force fields. From the CCS analysis, which reports on the global shape characteristics, local structural differences between individual aggregate conformers can be only partially resolved. To study the conformational changes towards ordered *β*-sheet structures, we further investigated the atomistic structures of the otherwise heterogeneous aggregate ensembles by integrating additional structural data.

### Probing the local inter-molecular substructure in the ensemble of low-molecular-weight oligomers

**Figure 6:**
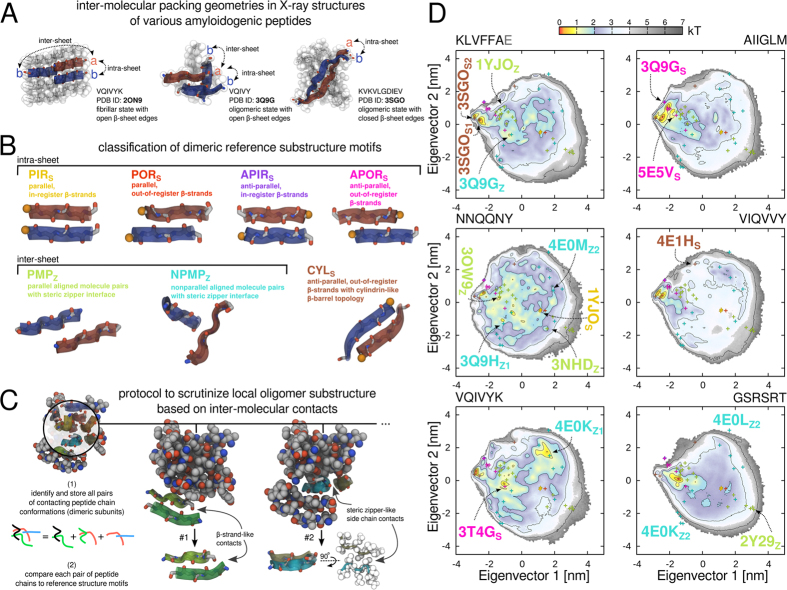
Structural ordering on the inter-molecular level and PCA projections of hexapeptide substructure conformations. (**A**) Inter-molecular packing geometries of amyloidogenic peptide molecules in selected aggregate states deposited in the PDB. (**B**) Classification of dimeric intra- and inter-sheet structure motifs as found in atomic models of amyloidogenic segments in the PDB. A colour scheme is used throughout the paper to distinguish the seven different substructure motifs. (**C**) Schematic example of decomposing a spontaneously assembled oligomeric aggregate from the simulations into pairs of closely contacting molecules. (**D**) PCA projections are shown as density plots. Colored circles denote location of reference structures from the PDB.

For a closer examination of the local substructure in the oligomeric aggregates obtained by our simulations, we next compared their inter-molecular packing geometry with the various atomic models of fibrillar^20^,^22^,^31^ and non-fibrillar assemblies^30^,^33^,^37^ of amyloidogenic peptide segments deposited in the Protein Data Bank (PDB, www.pdb.org)^68^ (Fig. 6A).

To find out if and which ordered conformations are accessible to amyloidogenic peptides in the early stages of oligomerization, we gathered a set of 44 non-redundant *β*-sheet motifs from structures of various amyloidogenic segments available in the PDB, which display substantial variations in their sequence (see Methods, Supplementary Methods and Supplementary Table 3).

More specifically, the set of experimentally determined dimeric reference substructures contains closely contacting hexapeptide molecule pairs and shows two characteristic configurations: edge-to-edge hydrogen-bonding interactions between strands (intra-sheet) and side-chain interactions between molecules of opposing *β*-sheet layers (inter-sheet)^1^,^20^,^69^ (Fig. 6A).

Figure 6B furthermore shows that the reference substructure motifs which feature main-chain hydrogen bonding interactions can be systematically discriminated according to their either parallel or anti-parallel *β*-strand orientations, as well as their small center-of-mass separation (Supplementary Fig. 7). The molecular pair structures with side-chain mediated contacts show a larger conformational variability due to the different inter-sheet spacings in the various steric zipper crystal structures. These structures also differ distinctly in terms of their varying strand crossing angle degree (0° to 180°, Supplementary Fig. 7). We grouped all types of dimeric hydrogen-bonded conformations into five intra-sheet structure motifs and labeled them PDB ID_S_ for further reference: anti-parallel *β*-strands with either in-register (APIR_S_) or out-of-register alignment (APOR_S_, CYL_S_) and parallel *β*-strand conformations with either in-register (PIR_S_) or out-of-register alignment (POR_S_). All dimeric peptide conformations with a tightly packed side-chain interface were grouped into two inter-sheet structure motifs and were labeled PDB ID_Z_: parallel molecule pairs (PMP_Z_) as found in different steric zipper classes with regular cross-*β* geometry and non-parallel molecule pairs (NPMP_Z_), which exhibit nonzero strand crossing angles (Fig. 6B).

To consistently compare the conformations of pre-fibrillar aggregates independent of oligomerization state with the seven basic motifs from the crystal structures, we extracted all contacting, non-redundant molecule pairs from the aggregates (dimers to dodecamers, *N* 2–12) spontaneously sampled in the simulations (see Methods, Fig. 6C). The obtained dimeric hexapeptide main-chain conformations were subsequently pooled and subjected to a principal component analysis (PCA)^44^,^70^ in order to gain insight into the local structural organization of the early oligomeric aggregates (see Methods, Fig. 6C,D). The PCA projections in Fig. 6D are a consensus over all used force fields and were obtained by using a common reference frame. This approach allows a direct comparison between the oligomeric substructure conformations of the studied sequences and with the set of reference motifs extracted from the PDB (see Methods and Supplementary Methods for details). The two main components of the covariance matrix of atomic displacements accounted for 45.9% of the total variance in the conformational space.

From the projections along the first two eigenvectors one can clearly distinguish the diversity of adopted conformational states (Fig. 6D). The PCA projections reveal several densely sampled conformational states (colored orange and yellow in Fig. 6D) common to all the amyloidogenic sequences. With the exception of NNQQNY, the overall most abundant dimeric structure motifs were out-of-register, anti-parallel *β*-strands similar to two cylindrin-like main-chain conformations from the PDB reference structures^31^,^37^ (PDB ID 3SGO in Fig. 6A, brown symbols in Fig. 6D) with either strong (3SGO_*S*1_) or weak (3SGO_*S*2_) hydrogen bonding interfaces.

Moreover, the aggregates of the amyloidogenic peptides with polar or charged amino-acids (KLVFFAE, NNQQNY and VIQVYK) showed additional prominent regions in the projections (colored yellow and cyan in Fig. 6D), which were not present for the hydrophobic peptides with predominantly aliphatic side chains (AIIGLM and VIQVVY). For example the NNQQNY aggregates consisted of a wide variety of substructure motifs close to known reference conformations, such as parallel in-register *β*-strands, as well as molecule pairs with a steric zipper interface at various strand crossing angles. The locations of selected reference structures in the PCA projections are highlighted in Fig. 6D and include PDB ID 4E0M, 3NHD, 3Q9H and 3OW9. Solid-state reference structures representing inter-sheet motifs with large strand crossing angles and large strand separation were not or only to a small extent covered by the simulation ensembles. These substructure elements may be energetically unfavorable in the solution-state due to exposed hydrophobic surface patches, as well as missing stabilizing inter-molecular contacts only present in the crystalline lattice^30^,^33^. Steric zipper structures with cross-*β* geometry and parallel strand orientations (e.g. PDB ID 2Y29 in the lower right of the PCA projection, Fig. 6D) were also not visited extensively, most likely due to the like-charge pairing of the peptide termini across the side-chain interface (Fig. 6D). The distributions of the substructure conformations showed multiple prominently sampled as well as broadened regions, which are distant from the set of experimentally determined structures (Fig. 6D).

Representative structures obtained by conformational clustering from these regions of the projected simulation structure ensembles reveal partially disordered aggregate substructure conformations (Supplementary Fig. 8). All studied sequences sampled abundant peptide molecule pair conformations, where either one or both chains were disordered, only partially extended or fully collapsed. Another group of conformations showed molecule pairs with only marginal contacts via terminal groups or backbone atoms (Supplementary Fig. 8). These conformations represent a common structural motif and underscore the importance of generic electrostatic interactions between the peptide backbones and charged termini in pre-nucleated aggregate structures. It is likely that these states are metastable instead of being additional ordered conformations, not yet covered by the solid-state reference structure ensemble.

### Ordered intra- and inter-sheet *β*-structures emerge transiently in oligomeric states

**Figure 7:**
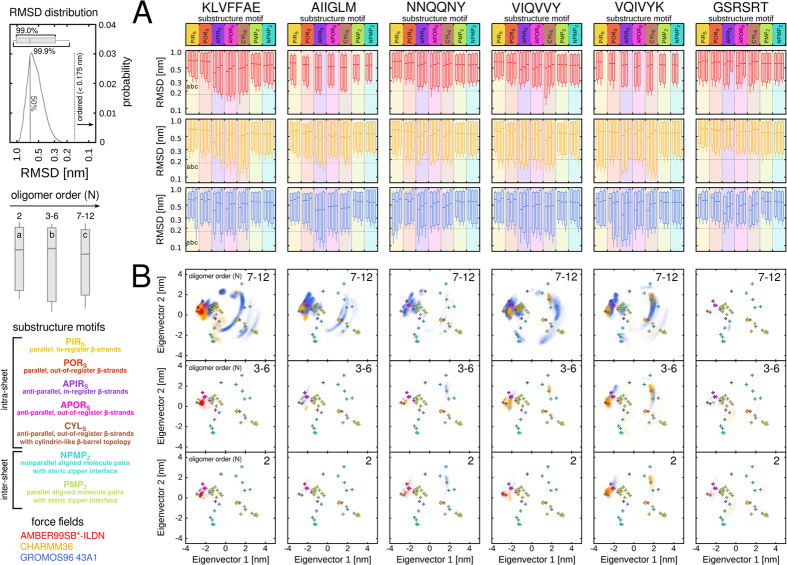
RMSD distributions of inter-molecular substructure conformations. (**A**) RMSD distributions of oligomeric substructure conformations with respect to sets of reference structure motifs are shown on a logarithmic scale as a function of oligomer size indicating 99.0% and 99.9% of the distribution widths (red, orange and blue box plots). (**B**) PCA projections summarize the occurrence of ordered substructure conformations (RMSD below 0.175 nm) as a function of the oligomerization state.

To further resolve the emergence and dynamics of sampling the ordered intra- and inter-sheet substructure conformations compatible with the PDB reference structure motifs, we carried out a RMSD distribution analysis. For each of the studied peptide sequences, RMSD distributions for dimeric substructure conformations were obtained from the ensemble of oligomeric states with respect to the available set of PDB structures per reference motif (Fig. 7A). The RMSD distributions are presented as box plots indicating the distribution widths enclosing 99.0% and 99.9% of the data, as well as the location of the median RMSD value (Fig. 7A). We pooled the RMSD distributions for the dimeric substructure conformations extracted from oligomeric states of size *N* 2, 3–6 and 7–12 for each sequence and force field combination to reduce the complexity of the presentation (Fig. 7A). From the median and width of the RMSD distributions it is apparent that the majority of the local substructure conformations extracted from the spontaneously sampled oligomers were not compatible with any of the seven experimentally determined PDB reference motifs (Fig. 7A). Even when considering several incremental RMSD cut-off criteria between 0.175 and 0.225 nm the fraction of disordered local substructures was relatively high (>90%), however, decreased with increasing oligomer size (Supplementary Fig. 9).

Figure 7B shows PCA projections of only the particularly ordered substructure conformations belonging to any of the seven motif categories (RMSD below 0.175 nm) as a function of oligomerization state. Although we observed the spontaneous population of smaller oligomers, the substructure of the non-amyloidogenic GSRSRT peptide aggregates of various sizes did not contain ordered conformations compatible with *β*-structure (Fig. 7A,B). For the remaining five amyloidogenic peptide systems in the current study, the RMSD distributions show an increased sampling of particularly ordered structures for almost all probed reference motifs with increasing oligomer size *N*, given that a reasonable sampling of the individual aggregation states was obtained (>100 ns, Fig. 2B). The observed substructure conformations include multiple different strand alignments, registries and facial packings within the hydrogen-bonded intra-sheet dimers, as well as rotational orientations of inter-sheet *β*-strand interfaces (Fig. 7A,B).

The RMSD distributions indicate that the oligomeric aggregates of most amyloidogenic sequences have substructure conformations with a particular preference for ordered, anti-parallel strand orientations (Fig. 7A,B). Smaller aggregates with ordered, parallel in-register *β*-strands were found only in case of the VQIVYK sequence (Fig. 7A,B), whereas ordered parallel *β*-strand substructures got more favorable in the larger KLVFFAE, NNQQNY and VIQVVY oligomers due to the increased probability in higher-order oligomers for larger *β*-sheet sizes to occur (Supplementary Fig. 4).

### Structure elements compatible with toxic precursors form prior to fibril-like conformations

We furthermore found consistent trends for the molecular level sheet-to-sheet recognition in the oligomers, although the individual higher-order oligomeric states (*N* ≥ 5) were populated in the simulations with different force fields to a varying extent. Substructure conformations with interdigitating side chains across a *β*-sheet interface and parallel as well as non-parallel *β*-strand geometries were found to emerge in the higher-order oligomeric aggregates of several amyloidogenic sequences. The present results suggest that transient non-fibrillar and fibrillar steric zipper structure elements are common motifs in higher-order oligomeric aggregates (Figs 6D and 7A,B).

**Figure 8:**
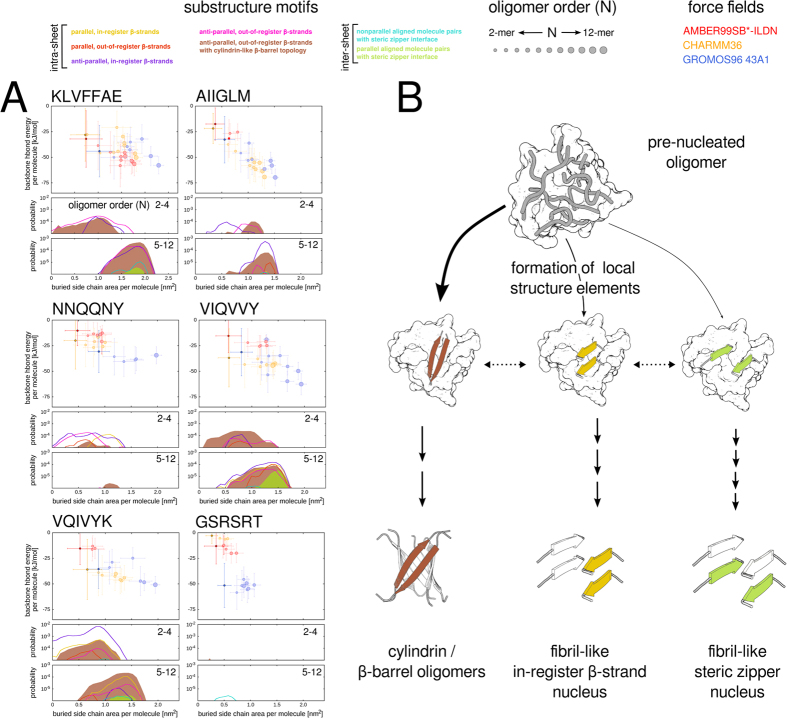
Occurrence of ordered inter-molecular substructure conformations during the pre-nucleation phase. (**A**) The upper parts of the panels show the inter-peptide backbone hydrogen bond energy and buried side-chain area per molecule for each oligomeric state in the three different force fields. The colored circles and corresponding bars represent the averages and standard deviations. Results for isolated dimers are highlighted by filled circles with bright colors and black outline. The lower parts of the panels summarize the occurrence of ordered substructure conformations as a function of the buried side-chain area per molecule and oligomerization state using the color code introduced in Fig. 6B. (**B**) Schematic illustration of the conformational sampling during the pre-nucleation phase of amyloidogenic peptide oligomerization. The aggregates oligomeric state encompassing anti-parallel, out-of-register *β*-strands compatible with *β*-barrel oligomers, as well as fibril-like structural elements.

Figure 8A depicts the occurrence of defined *β*-strand and *β*-sheet-to-*β*-sheet packing motifs as a function of buried side-chain surface area for different sampled oligomeric states and illustrates that the presence of steric zipper-like substructure conformations overall correlates with the increased peptide side-chain burial in the higher-order oligomeric states compared to the isolated dimer and smaller aggregates. We conclude that pre-fibrillar solution-state aggregates of amyloidogenic sequences exclude large fractions of their molecular surface area from the solvent by creating dry interfaces between adjacent *β*-sheets. The observed interface conformations are packed together via close side-chain contacts and exhibit high shape complementarities (sc) comparable to known steric zipper X-ray crystal structures (sc: 0.65–0.85)^20^,^30^,^31^. The extent of side-chain burial was found to be closely related to the strength of the main-chain hydrogen-bonding interactions, as can be seen by the averages over all aggregates with ordered substructure conformations per oligomeric state (Fig. 8A). This finding indicates a cooperative formation of hydrogen-bonded strands and directed side-chain interactions between opposing *β*-sheet units in higher-order peptide oligomers and suggests an overall intricate interplay between specific combinations of intra- and inter-sheet *β*-strand conformations. Moreover, the observed sequestering of molecular surface facilitates the aggregate substructure ordering and the formation of stable *β*-sheet-to-*β*-sheet packing motifs with a regular cross-*β* geometry, which have been deemed critical for fibrillar growth^35^,^41^,^69^.

Accumulating knowledge of X-ray structures from various peptide aggregation states^20^,^22^,^30^,^31^,^37^,^71^ led to two diverging aggregation pathway proposals for amyloid forming peptides and proteins. On the molecular level, both pathways feature a very distinct organization in the structural elements of either elongated sheets of in-register *β*-strands (fibrils) or anti-parallel, out-of-register *β*-strands in a closed *β*-barrel oligomer. The latter species has been found to exhibit elevated cytotoxicity levels^31^,^37^, but was shown to convert into in-register fibril structures eventually.

Most notably, our simulations show that the specific out-of-register *β*-strand motifs, known to constitute toxic oligomers with characteristic *β*-barrel folds, were present in aggregates as small as the dimer and commonly found for all amyloidogenic peptide sequences. This result suggests that the formation of building blocks for potentially toxic aggregate conformations is favored in the pre-nucleation phase over the fibril-like substructure elements, which appear only after the assembly of larger peptide aggregates (Fig. 8A,B).

### Diverse fibril-like conformations in the ensemble of oligomeric aggregates highlight the structural complexity of the pre-fibrillar state

Our simulations indicate that amyloidogenic peptide oligomers transiently sample a multitude of ordered *β*-structure elements already early along the aggregation pathway. As an illustrative example to scrutinize the implications of sampling ordered aggregate substructures in pre-fibrillar oligomers, we chose to look into the simulations of the KLVFFAE segment of the amyloid-*β* peptide. In contrast to most known fibrillar models of peptides and proteins^19^,^31^,^69^,^72^, as well as the AIIGLM, NNQQNY and VQIVYK steric zipper crystal structures^20^ that uniformly display a parallel in-register strand arrangement, KLVFFAE exhibits both a parallel and anti-parallel stranded *β*-sheet structure in the fibrillar state.

**Figure 9:**
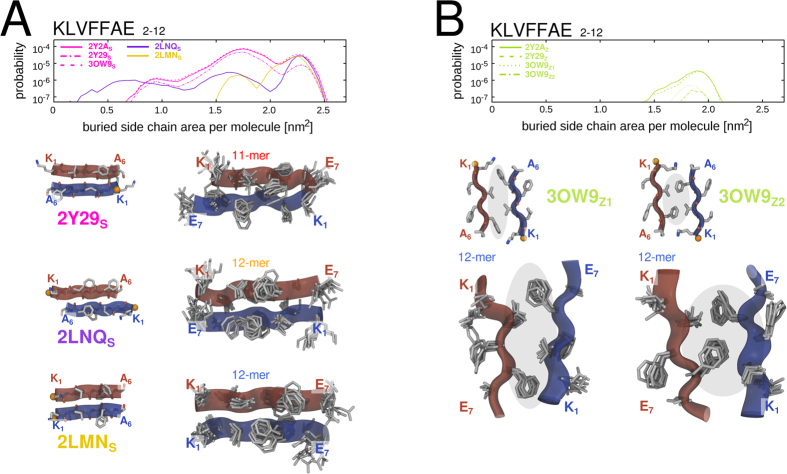
Comparison of KLVFFAE substructure conformations to polymorphic fibrillar reference structures from the PDB. (**A**) Histograms of ordered intra- and (**B**) inter-sheet KLVFFAE substructure conformations (main-chain and C_*β*_ atomic coordinates) are shown an a function of buried side-chain area. Representative ensembles of ordered substructure conformations (KLVFFAE) from simulations in several force fields are shown next to the corresponding fibrillar reference structures (KLVFFA) from the PDB.

Figure 9 presents multiple ordered intra- and inter-sheet substructure motifs observed in the higher-order oligomeric states of KLVFFAE. The shown substructures are compatible with several structurally diverse reference conformations for which polymorphic cross-*β* structures were experimentally determined. Note that specific side-chain orientations were taken explicitly into account, such that sub-ensemble with distinct side-chain packing modes could be separated. The structural complexity found for the pre-fibrillar oligomeric states thus offers an intriguing clue to elucidate the origin of molecular polymorphism observed in mature amyloid aggregates such as high-molecular-weight oligomers, fibrils and crystals^1^,^18^,^19^,^25^,^69^,^73^.

## Discussion

All-atom explicit solvent MD simulations were carried out to investigate the amyloidogenic peptide oligomerization process on the *μ*s time scale. A consensus force field approach enabled us to extensively sample individual aggregation states and revealed the molecular events underlying the initial stages of peptide oligomer self-assembly. To this end, we combined the simulation results in multiple MD force fields to minimize a potential bias imposed by a single force field parameter set. Our study indicates that the dynamics and aggregation propensities differ for the tested force fields with a delicate dependence on sequence and simulation time scales. The intrinsic secondary structure propensity, polypeptide chain dimensions in the unfolded state and non-covalent interactions that govern molecular binding and recognition have been in focus of recent efforts to assess and improve the performance of MD force fields^74^,^75^,^76^,^77^. We conclude that evaluating the accurate description of the conformational states relevant to inter-molecular self-assembly also warrants further validation of empirical force field parameter quality. The consensus force field analysis presented here, however, suggests that current non-polarizable force fields are able to capture the global features of the peptide oligomerization process and to discriminate between aggregation-prone and non-fibrilizing sequences.

Along these lines, our simulation results show that the spontaneous population of higher-order oligomeric states took place on the sub-*μ*s time scale in non-dilute solutions and was preceded by a dynamic agglomeration phase during the simulated oligomerization process. Despite the presence of multiple, mainly transient low-molecular-weight oligomers, a significant fraction of the pre-nucleated oligomers displayed a lattice-like *β*-structure. In agreement with experimental and theoretical evidence, the high degree and early onset of oligomerization found for the peptides with hydrophobic residues stresses the importance of hydrophobicity as a major molecular driving force early in the self-assembly process^35^,^38^,^45^,^78^.

The population of ordered *β*-sheet structure was related to the spontaneous oligomer growth process during the simulations in a similar manner as previously described for simulations of the early stages of amyloidogenic peptide oligomerization^44^,^48^,^53^.

Conformations with high *β*-sheet content were present to a significant extent already within the dimeric to tetrameric aggregates for KLVFFAE, VQIVYK and VIQVVY, similar to other short peptides exhibiting early and profound conformational conversions toward the *β*-state^34^,^65^.

Structural details obtained from CCS ensemble distributions of individual oligomeric states showed that the quaternary structure of low-molecular-weight oligomers is consistent with several lines of evidence for the proposed self-assembly pathway toward *β*-structured aggregates^6^,^30^,^34^,^35^,^65^. Firstly, the CCS analysis showed that the simulated spontaneous oligomerization process yields a diverse ensemble of solution-state conformations. Early oligomeric aggregates displayed a rather dynamic and heterogeneous, opposed to a single defined quaternary structure. Secondly, we found predominately aggregates that exhibited a significant amount of lattice-like *β*-structure partitioned in several smaller *β*-sheet subunits and arranged as multi-layers instead to single elongated *β*-sheet filaments. This also establishes a relationship between the presence of ordered *β*-sheet structure within the oligomeric aggregates and the global aggregate shape, which deviated strongly from isotropic growth in all of the individual oligomeric states. Our findings are in line with X-ray and ssNMR data on pre-fibrillar oligomers^26^,^28^,^30^,^33^,^36^,^61^,^71^,^73^ and previous MD simulations of short C-terminal A*β* peptides, which estimated a low free energy for aggregates composed of two and three stranded *β*-sheets^46^,^50^,^54^,^59^. Moreover in accordance with theoretical studies on *β*-sheet aggregates^45^,^58^, we found that pre-nucleated oligomers achieve structural stability by adopting compact layered conformations, thereby optimizing the number of favorable inter-molecule contacts between the peptide chains and shielding backbone-backbone hydrogen bonds from the solvent.

To gain insight into the local structural organization of the early aggregates, we probed the oligomeric state for common structure motifs previously reported by solid-state experiments. A multitude of intra- and inter-sheet structural elements known from mature fibrillar and non-fibrilliar aggregates was transiently visited: In line with previous theoretical studies^44^,^46^ on the oligomerization of small fragments from amyloidogenic peptides employing a less strict contact criterion to define intra-sheet *β*-strand interactions, we found that mixed, parallel/anti-parallel stranded *β*-sheet structures dominate over fully parallel or anti-parallel orientations with increasing *β*-sheet size. Oligomers with mixed *β*-strand polarities and out-of-register strands different from the structural organization of the fibrillar state are also concurrent with experimental observations made for conformationally constrained peptide model systems^30^,^33^, as well as stable conformations of oligomers from the full-length amyloid-*β* peptide^25^,^27^,^28^,^29^ and *α*-synuclein^26^. The wide variety of ordered substructure conformations observed in the simulations, furthermore helps to rationalize the seemingly complex surface structure of pre-fibrillar oligomers as determined by conformation specific antibodies^1^,^9^,^79^.

The formation of higher-order oligomeric states for several different amyloidogenic sequences proceeded with a noticeable build-up of self-complementary interfaces between adjacent *β*-sheets. This finding hints at layered *β*-sheet motifs being a transient feature of the oligomeric state in solution, sampling similar steric zipper *β*-sheet structures earlier observed in X-ray and ssNMR structures of oligomerized model constructs in the solid-state^28^,^30^,^33^. The example of GSRSRT peptide aggregates nicely showed that the structural complementarity of side-chain interfaces and favorable *β*-strand interactions are required for the formation of higher-order oligomer aggregates and their structural ordering during the oligomerization process^39^. The arginine side-chains contained in the sequence of the peptide are known to cause clashes in a fibrillar steric zipper spine structure with close-by *β*-strands along the axis of hydrogen-bonding within the same *β*-sheet and to adjacent *β*-sheet layers^80^,^81^. Consequently, GSRSRT did not populate higher-order aggregates and the substructure of GSRSRT oligomers was dominated by only a single disordered *β*-strand conformation not located close to any of the PDB reference structure motifs. In summary, it is thus very likely that the dry *β*-sheet interfaces represent common packing motifs in the oligomerization phase of amyloidogenic peptides at the atomic level, whereas the homogeneity and stability of *β*-sheet substructure conformations determines the probability of fibril formation.

Moreover, our findings illustrate that the aggregated oligomeric state of amyloidogenic sequence segments facilitates the conversion to ordered fibril-like molecule conformations despite the fact that the majority of substructures was found to be at least partially disordered. Although we cannot entirely construe the whole range of interaction patterns for the ensemble of disordered aggregate substructures and their energetic contributions to the gradual and cooperative structural transitions of the oligomers, a recent theoretical study has pointed out that also nonspecific interactions within small oligomers indeed play a major role in stabilizing the aggregates and are crucial for the nucleation in the regime of low peptide concentrations^82^. Along these lines and in agreement with previous studies, our findings suggest that partially disordered, metastable states in the self-assembly process are prevalent and longer-lived for hydrophilic sequences compared to hydrophobic ones, which are more prone to sample *β*-competent conformations^20^,^42^,^45^. At the same time, we found that the formation of ordered building blocks of potentially toxic *β*-barrel aggregates is favored early on in the oligomerization process, indicating that those structures directly relate to the pre-nucleation phase of fibrillization.

## Methods

### MD simulations and consensus force field approach

The GROMACS simulation package (version 4.5.5 and 4.6^83^,^84^) was used to set up, carry out and analyze the MD simulations. Seven biomolecular force fields and three different water models were used to benchmark and cross-reference the simulation outcomes. The following force fields were employed: AMBER99SB*-ILDN^85^,^86^,^87^, AMBER03^62^, CHARMM22* 88 and CHARMM36^74^,^89^ were used together with the TIP3P water model^90^, the GROMOS96 43A1^91^ and GROMOS96 54A7^92^ force field along with the SPC water model^93^ and OPLS-AA/L^94^,^95^ with the TIP4P water model^90^.

The AMBER03 and GROMOS96 force fields have been widely used to study *in sillico* aggregation systems in the past^44^,^45^,^57^,^60^,^96^. To validate the performance of current non-polarizable force fields with explicitly represented solvent molecules and to minimize a possible force field bias, we also included the AMBER99SB*-ILDN and CHARMM36. These two refined variants of popular MD force fields are the result of very recent efforts to balance secondary structure propensities^74^,^85^,^86^,^87^,^97^, as well as to decrease the compactness of the unfolded state^74^,^75^. In all simulations, the Particle-Mesh-Ewald (PME)^98^,^99^ method to treat long-ranged electrostatic interactions was applied together with periodic boundary conditions to the simulation box. A full description of the simulation settings is given in the Supplementary Methods Section.

### Simulation setup

The protocol with which all the simulation systems were set up is detailed in the Supplementary Methods Section. Simulations of 12 peptide molecules in cubic boxes with edge lengths of 10.0 nm and 21.5 nm were initiated from fully randomized monomeric conformations (Fig. 1). All simulation systems were solvated with explicit water molecules resulting in a final peptide concentration of 20 mM and 2 mM, respectively (Table 1).

Lysine, arginine and glutamate residues, as well as the peptides’ N- and C-termini were simulated in their charged states. Counter-ions (Na^+^, Cl^−^) were added to yield an appropriate ionic strength (0.15 M) and to neutralize the net system charge. An energy minimization using steepest descent was performed prior to starting the simulations.

### Data analysis

From the individual simulation trajectories samples were taken for all steps of subsequent analyses every 10 ps. A pairwise contact analysis was carried out to identify all individual oligomer sizes ranging from 2-mer to 12-mer after pooling all trajectories of each respective peptide sequence and force field combination: peptides that share an inter-chain residue contact are counted to be within the same aggregate. For any two residues *i*, *j*, an inter-chain contact is considered as formed, if any heavy atom of residue *i* is within a cutoff of 0.35 nm from any heavy atom of residue *j*.

### Extent of oligomerization

The specific association state of the peptide molecules was described to quantify the presence of higher-order oligomeric states. For each trajectory frame, the sum over the square roots of all present aggregate sizes was calculated as 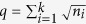, with *k* as the total number of aggregates and *n* as the number of peptides in aggregate *i*. Subsequently, *q* was normalized against *N*, as the total number of peptides in the simulation box (*N* = 12), such that 
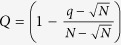
. *Q* reports as a convoluted, yet unambiguous measure on the fractional population of each aggregate size ranging from 1 to 12 and thus, on the overall oligomerization progress from a fully monomeric (*Q* = 0) to a fully oligomerized state (*Q* = 1)^45^,^47^,^100^.

### Calculation of collision cross sections

For every combination of sequence, force field and oligomer size *N* = 2–12, up to 1000 structures were randomly selected for the calculation of collision cross sections (CCSs). In case fewer than 1000 simulation snapshots were available, all sampled structures for this combination were used. Subsequently, the selected structures were energy minimized in vacuum using the steepest descent algorithm^83^,^84^. The CCS of the minimized structures was calculated using the Trajectory method^67^ as implemented in the MOBCAL software. For the CCS calculations, charges native to the force field in which the simulations had been carried out were retained. The CCS was estimated for every structure individually by calculating 500k trajectories. Anisotropic theoretical reference values were computed without energy minimization by single point calculations of ideal single and double layer *β*-sheet structures modeled based on the coordinates of the X-ray crystal conformation of the amyloidogenic peptides^20^,^22^,^34^.

Reference CCSs for isotropic structures were calculated based on the empirical formula *σ*_*N*_ = *σ*_1, exp._ ⋅ *N*^2/3^ 34. If no experimental value was available, the respective monomer CCS was obtained from the simulations, as the consensus average over 1000 randomly selected random coil conformations from simulation ensembles of all three force fields (*σ*_1, sim._).

### Prediction of CCSs with Partial Least Squares regression

CCSs for the whole set of structures obtained by MD simulations were predicted using Partial Least Squares (PLS) regression models^101^. The set of CCS values calculated by means of the Trajectory method was used as a dependent variable for the regression model building. Nine descriptors (i. a. hydrogen bond energies, radius of gyration) were used as independent regressors (see Supplementary Methods for details). For each sequence, force field and oligomer size combination, a PLS model was built independently based on the respective structure ensemble for which CCS values were calculated: half of the structures were randomly assigned to the “training” group, whereas the rest of the structures were left for cross-validation. Monitoring the change in the correlation coefficients between the calculated and the predicted CCS values revealed that three PLS components provided sufficient accuracy avoiding an overfitting problem. To evaluate the quality of the PLS models, correlation coefficients and average unsigned errors (AUE) between the predicted values and the CCS estimates from the Trajectory method in the cross-validation sets were calculated.

### Decomposing oligomeric structures into ensembles of molecule pairs

To study the substructure of each identified oligomer of size *N* for every sequence and force field, the following approach of decomposition into pairs of peptide chains was used: for each oligomeric structure, all possible peptide chain pairs that share at least one heavy atom contact within a distance of 0.35 nm were stored^44^. Only unique molecule pair combinations were considered by removing identical and permuted combinations, e.g. molecule pairs *AB* and *BA* were treated as equivalent and one of them was discarded. A conformational ensemble of dimeric structures was obtained and pooled separately for each specific oligomeric state consisting of *N* peptides (where *N* = 2–12). Subsequently, an iterative relabeling scheme was applied such that structurally similar molecule pairs were given the same chain identifiers (ID_S_). This relabeling allows for a consistent superposition to a reference structure using the variance minimization method^102^. Main-chain atoms were used for fitting and the root-mean-square deviation (RMSD) calculation. To fit the requirement of a hexapeptide segment, we selected the KLVFFA stretch from the ^16^KLVFFAE^22^ peptide for further analysis.

### Dimeric reference structure motifs

Atomic coordinates of fibrillar and non-fibrillar assemblies of amyloidogenic hexapeptide segments were downloaded from the Protein Data Bank (PDB, www.pdb.org)^68^ and a database containing predictions of fibril-forming segments (ZipperDB, http://services.mbi.ucla.edu/zipperdb/)^40^. For each structure model, dimeric intra- and inter-sheet main-chain coordinates were derived as representative molecular pair reference structures with a common subset of protein heavy atoms (Supplementary Table 3).

### Principal component analysis of oligomeric substructure conformations

A principal component analysis (PCA)^70^ was carried out on the main-chain atomic coordinates of each molecule pair structure ensemble obtained from the decomposition of the spontaneously formed oligomers (2-mer to 12-mer) for each sequence and force field. All the oligomeric substructure ensembles for each sequence and oligomeric state were projected onto a common reference frame, namely a consensus set of eigenvectors (1st ev and 2nd ev). The eigenvectors used for the projections were derived over all of the dimeric PDB reference structures and a particularly ordered sub-ensemble of structures obtained from the MD simulations of all sequences and all force fields (using a RMSD cut-off criterion of 0.2 nm to any of the reference structures). Prior to the construction and diagonalization of the covariance matrix of atomic displacements, all reference conformations were superimposed using the variance minimization method^102^. To obtain representative structures of the projected ensembles, a conformational clustering was performed using the k-means Hartigan-Wong algorithm^103^ implemented in the statistical software package R^104^. Cluster centers were selected according to the global k-means algorithm^105^. Structures with the lowest RMSD value to the geometrical centers of the clusters were selected as representatives.

## Additional Information

**How to cite this article**: Matthes, D. *et al*. An Atomistic View of Amyloidogenic Self-assembly: Structure and Dynamics of Heterogeneous Conformational States in the Pre-nucleation Phase. *Sci. Rep.* **6**, 33156; doi: 10.1038/srep33156 (2016).

## Supplementary Material

Supplementary Information

## Figures and Tables

**Table 1:** Summary of performed simulations starting from random monomeric states.

Sequence	Size of system	Force Field	Length of simulations
Amyloid *β*_16–22_	100 Å × 100 Å × 100 Å;	AMBER99SB*-ILDN	2.24 *μ*s, 2.09 *μ*s, 1.89 *μ*s
KLVFFAE	12 peptide molecules;	CHARMM36	1.27 *μ*s, 1.15 *μ*s, 1.12 *μ*s
(20 mM)	~32.400 water molecules;	GROMOS96 43A1	1.37 *μ*s, 1.36 *μ*s, 1.23 *μ*s
	~100.000 total atoms		
Amyloid *β*_16–22_	215 Å × 215 Å × 215 Å;	AMBER99SB*-ILDN	2 × 0.10 *μ*s
KLVFFAE	12 peptide molecules;	CHARMM36	2 × 0.10 *μ*s, 0.05 *μ*s
(2 mM)	~331.000 water molecules;	GROMOS96 43A1	0.10 *μ*s, 2 × 0.05 *μ*s
	~1.000.000 total atoms		
Amyloid *β*_30–35_	100 Å × 100 Å × 100 Å;	AMBER99SB*-ILDN	1.35 *μ*s, 1.33 *μ*s, 1.28 *μ*s
AIIGLM	12 peptide molecules;	CHARMM36	1.14 *μ*s, 1.12 *μ*s, 1.07 *μ*s
(20 mM)	~32.600 water molecules;	GROMOS96 43A1	1.24 *μ*s, 1.14 *μ*s, 1.13 *μ*s
	~100.000 total atoms		
Amyloid*β*_30–35_	215 Å × 215 Å × 215 Å;	AMBER99SB*-ILDN	0.05 *μ*s
AIIGLM	12 peptide molecules;	CHARMM36	0.10 *μ*s, 2 × 0.05 *μ*s
(2 mM)	~331.200 water molecules;	GROMOS96 43A1	2 × 0.10 *μ*s, 0.05 *μ*s
	~1.000.000 total atoms		
Sup35p_8–13,wt_	100 Å × 100 Å × 100 Å;	AMBER99SB*-ILDN	1.38 *μ*s, 1.31 *μ*s
NNQQNY	12 peptide molecules;	CHARMM36	1.14 *μ*s, 1.09 *μ*s
(20 mM)	~32.600 water molecules;	GROMOS96 43A1	1.03 *μ*s, 0.99 *μ*s, 0.29 *μ*s, 0.27 *μ*s
	~100.000 total atoms		
Sup35p_8–13,wt_	215 Å × 215 Å × 215 Å;	AMBER99SB*-ILDN	0.1 *μ*s
NNQQNY	12 peptide molecules;	CHARMM36	0.1 *μ*s
(2 mM)	~331.200 water molecules;	GROMOS96 43A1	2 × 0.1 *μ*s
	~1.000.000 total atoms		
Sup35p_8–13,mut_	100 Å × 100 Å × 100 Å;	AMBER99SB*-ILDN	1.37 *μ*s, 1.33 *μ*s
VIQVVY	12 peptide molecules;	CHARMM36	1.30 *μ*s, 1.05 *μ*s
(20 mM)	~32.600 water molecules;	GROMOS96 43A1	0.97 *μ*s, 0.96 *μ*s, 0.19 *μ*s
	~100.000 total atoms		
Sup35p_8–13,mut_	215 Å × 215 Å × 215 Å;	AMBER99SB*-ILDN	0.05 *μ*s
VIQVVY	12 peptide molecules;	CHARMM36	0.05 *μ*s
(2 mM)	~331.200 water molecules;	GROMOS96 43A1	0.1 *μ*s
	~1.000.000 total atoms		
hTau40_306–311_	100 Å × 100 Å × 100 Å;	AMBER99SB*-ILDN	1.46 *μ*s, 1.44 *μ*s, 1.26 *μ*s
VQIVYK	12 peptide molecules;	CHARMM36	1.24 *μ*s, 1.03 *μ*s, 1.03 *μ*s, 0.95 *μ*s
(20 mM)	~32.500 water molecules;	GROMOS96 43A1	1.15 *μ*s, 1.12 *μ*s, 1.09 *μ*s
	~100.000 total atoms		
hTau40_306–311_	215 Å × 215 Å × 215 Å;	AMBER99SB*-ILDN	0.05 *μ*s
VQIVYK	12 peptide molecules;	CHARMM36	0.10 *μ*s, 0.05 *μ*s
(2 mM)	~331.200 water molecules;	GROMOS96 43A1	3 × 0.10 *μ*s
	~1.000.000 total atoms		
hTau40_307–212_	100 Å × 100 Å × 100 Å;	AMBER99SB*-ILDN	2.50 *μ*s, 1.58 *μ*s
GSRSRT	12 peptide molecules;	CHARMM36	2.00 *μ*s, 1.50 *μ*s
(20 mM)	~32.600 water molecules;	GROMOS96 43A1	2.00 *μ*s, 2.00 *μ*s
	~100.000 total atoms		

For each studied sequence segment and employed force field, the length of each independent MD simulation is reported together with the total system size and number of simulated particles.
