# Supplementary material for: An Atomistic View of Amyloidogenic Self-assembly: Structure and Dynamics of Heterogeneous Conformational States in the Pre-nucleation Phase
Source: Sci Rep. 2016 Sep 12;6:33156. doi: 10.1038/srep33156 (PMC5018807; doi:10.1038/srep33156)
Supplement: Supplementary Information [file srep33156-s1.pdf]

Supplementary Information

An Atomistic View of Amyloidogenic Self-assembly: Structure and Dynamics of Heterogeneous Conformational States in the Pre-nucleation Phase

Dirk Matthes, Vytautas Gapsys, Julian T. Brennecke and Bert L. de Groot
Computational Biomolecular Dynamics Group, Max Planck Institute for Biophysical Chemistry, Goettingen, Germany

Supplementary Methods

MD simulation setup.

The starting configurations for the spontaneous oligomerization simulations with initially monomeric peptides were generated by placing 12 molecules randomly in position and orientation in a cubic box of $1000\,\mathrm{nm}^3$ (10x10x10 nm) and $10000\,\mathrm{nm}^3$ (21.5x21.5x21.5 nm). Simulations of pre-assembled β-oligomers were initiated from dodecameric single and double β-sheet structure models modelled based on crystalline structure coordinates. To construct these reference states, the atomic coordinates of the PDB entries 2Y3J (AIIGLM) (1), 2ON9 (VQIVYK), 1YJO (NNQQNY) (2), the coordinates of a modelled Amyloid-β fibre protofilament with steric zipper interfaces determined by X-ray crystallography were used (residues 16-22, KLVFFAE)(1). In cases where no experimental crystal structure was available (VIQVVY, GSRSRT), we used the structure model coordinates provided by the zipperDB database (VIQVVY, GSRSRT) (3), by replicating the asymmetric unit along the appropriate lattice axes.

Force field and MD settings.

In all simulations the velocity-rescaling (4) algorithm was applied to couple the simulation system to an external heat bath with a temperature of 300 K using a time constant of $\tau = 0.1\,\mathrm{ps}$. Initial velocities were taken according to a Maxwell-Boltzmann distribution at 300 K. All protein bonds were constrained using P-LINCS (5). Water molecules were constrained using SETTLE (6). The long-ranged electrostatic interactions were calculated by the Particle-Mesh-Ewald (PME) (7, 8) method at every step with a grid spacing of 0.1 nm. The relative tolerance at the cut-off was set to 10^{-6}. The following force field specific settings were applied:

AMBER99SB*-ILDN and AMBER03. Hydrogen atoms were converted to virtual sites (9) and the integration time-step was set to 4 fs. The neighbour lists for non-bonded interactions were updated every 3 steps. The short-ranged van der Waals and electrostatic interactions were cut-off at 1.0 nm. For pressure coupling the scheme of Parrinello-Rahman (10) was used to hold the system at a pressure of 1 bar (τ = 2.5 ps).
CHARMM36 and CHARMM22*. The integration time-step was set to 2 fs. The neighbour lists for non-bonded interactions were updated every 10 steps. The van der Waals interactions were switched off between 1.0 to 1.2 nm and short-ranged electrostatic interactions were cut-off at 1.2 nm. For pressure coupling the scheme of Parrinello-Rahman (10) was used to hold the system at a pressure of 1 bar (τ = 2.5 ps).
Additional simulations with the AMBER99SB*-ILDN and CHARMM36 force fields were performed with the native GPU accelerated version 4.6 of the GROMACS software package (11). A non-bonded Verlet scheme with cut-off for the van der Waals and electrostatic interactions together with a buffered pair-list was utilized, respectively. The integration time-step was set to 4 fs using virtual sites for hydrogen atoms.
GROMOS96 43A1 and GROMOS96 54A7. Virtual sites for hydrogen atoms were used to increase the integration time-step to 4 fs. The neighbour lists for non-bonded interactions were updated every 5 steps. The van der Waals and short-ranged electrostatic interactions were cut-off at 0.9 nm and 1.4 nm, respectively. The Berendsen coupling algorithm (12) was applied to keep the pressure constant by coupling the system to a pressure bath of 1 bar (τ = 1 ps).
OPLS-AA/L. The integration time-step was set to 4 fs. The neighbour lists for non-bonded interaction were updated every 5 steps. The van der Waals interactions and short-ranged electrostatic interactions were cut-off at 0.9 and 1.4 nm, respectively. For pressure coupling the scheme of Parrinello-Rahman (10) was used to hold the system at a pressure of 1 bar (τ = 2.5 ps). Virtual sites were used for hydrogen atoms.

PLS regression model building.

The following descriptors were used as independent regressors for the PLS model building:
1) Fractions of β-sheet content and 2) coil content; 3) the main-chain solvation free energy; 4) the side-chain solvation free energy; hydrogen bond energies: 5) intra-chain, 6) inter-chain side-chain to side-chain, 7) inter-chain side-chain to main-chain, 8) inter-chain main-chain to main-chain; 9) radius of gyration.

The independent variables were z-scored by pooling hydrogen bond energies, secondary structure elements, solvation free energies into separate groups and leaving radius of gyration in its own group.
The bulk of the analysis was carried out using GROMACS utility programs such as *g_sas*, *g_gyrate* and *do_dssp*. The solvation free energy as defined by Eisenberg and McLachlan (13) was computed using a solvent probe radius of 1.4 nm. The hydrogen bond energy was calculated according to Espinosa *et al.* (14) and decomposed into the contributions listed above.

Supplementary Tables

Table 1. Summary of additional simulations.

Sequence	Size of system	Force Field	Length of simulations [μs]
Amyloidβ_{16-22}	100 Å× 100 Å× 100 Å;	AMBER99SB*-ILDN	$0.13^{\dagger}$
KLVFFAE	12 peptide molecules;	CHARMM36	$2 \times 0.1^{\dagger}$
(20 mM)	~ 32.400 water molecules;	GROMOS96 43A1	$0.1^{\dagger}$
	~ 100.000 total atoms		
Amyloidβ_{30-35}	100 Å× 100 Å× 100 Å;	AMBER03	2×0.7
AIIGLM	12 peptide molecules;	AMBER99SB*-ILDN	$0.47^{\dagger}$, $2 \times 0.3^{\ddagger}$
(20 mM)	~ 32.600 water molecules;	CHARMM36	$3 \times 0.1^{\dagger}$, $2 \times 0.05^{\dagger}$, $0.3^{\ddagger}$, $0.26^{\ddagger}$
	~ 100.000 total atoms	GROMOS96 43A1	$0.1^{\dagger}$
		OPLS-AA/L	2×0.5
		GROMOS96 54A7	0.3
		CHARMM22*	2×0.5
Amyloidβ_{30-35}	100 Å× 100 Å× 100 Å;	AMBER99SB*-ILDN	2×0.5
Nme-AIIGLM-Ace	12 peptide molecules;		
(20 mM)	~ 32.600 water molecules;		
	~ 100.000 total atoms		
Amyloid$\beta_{30-35,\,\mathrm{mut}}$	100 Å× 100 Å× 100 Å;	AMBER99SB*-ILDN	0.5
AIIALM	12 peptide molecules;		
(20 mM)	~ 32.600 water molecules;		
	~ 100.000 total atoms		
Sup35p$_{8-13,\,\mathrm{wt}}$	100 Å× 100 Å× 100 Å;	AMBER03	1.39, 1.16
NNQQNY	12 peptide molecules;	AMBER99SB*-ILDN	$0.12^{\dagger}$
(20 mM)	~ 32.600 water molecules;	CHARMM36	$0.16^{\dagger}$, $0.12^{\dagger}$
	~ 100.000 total atoms	GROMOS96 43A1	$0.1^{\dagger}$
Sup35p$_{8-13,\,\mathrm{mut}}$	100 Å× 100 Å× 100 Å;	AMBER03	1.35, 1.1
VIQVVY	12 peptide molecules;	AMBER99SB*-ILDN	$0.13^{\dagger}$
(20 mM)	~ 32.600 water molecules;	CHARMM36	$2 \times 0.1^{\dagger}$
	~ 100.000 total atoms	GROMOS96 43A1	$0.9^{\dagger}$
hTau40$_{306-311}$	100 Å× 100 Å× 100 Å;	AMBER99SB*-ILDN	$0.09^{\dagger}$
VQIVYK	12 peptide molecules;	CHARMM36	$0.21^{\dagger}$, $2 \times 0.12^{\dagger}$
(20 mM)	~ 32.500 water molecules;	GROMOS96 43A1	$0.09^{\dagger}$
	~ 100.000 total atoms		
hTau40$_{307-212}$	100 Å× 100 Å× 100 Å;	AMBER03	1.01, 0.98
GSRSRT	12 peptide molecules;		
(20 mM)	~ 32.600 water molecules;		
	~ 100.000 total atoms		

Symbols denote simulations of pre-assembled β-oligomers modeled from reference crystal structure conformations (†) or snapshots of GROMOS96 43A1 trajectories (‡).

Table 2. Correlation coefficients of training and testing sets for the PLS models predicting the collision cross sections for each combination of sequence, force field and oligomer size N.

Sequence (force field) / N	2	3	4	5	6	7	8	9	10	11	12
KLVFFAE (AMBER99SB*-ILDN)	0.84/0.81	0.87/0.85	0.89/0.88	0.91/0.90	0.89/0.89	0.87/0.87	0.89/ 0.90	0.74/ 0.75	0.83/0.89	0.89/0.86	0.75/0.80
AIIGLM (AMBER99SB*-ILDN)	0.85/0.84	0.91/0.88	0.85/0.85	0.90/0.90	-/-	-/-	-/-	-/-	-/-	-/-	-/-
NNQQNY (AMBER99SB*-ILDN)	0.89/0.86	0.89/0.86	0.89/0.88	0.86/0.81	0.81/0.85	0.89/0.81	0.81/0.91	-/-	-/-	-/-	-/-
VIQVVY (AMBER99SB*-ILDN)	0.90/0.86	0.90/0.91	0.92/0.92	0.88/0.88	0.92/0.91	0.84/0.78	-/-	-/-	-/-	-/-	-/-
VQIVYK (AMBER99SB*-ILDN)	0.90/0.89	0.89/0.90	0.89/0.90	0.77/0.74	-/-	-/-	-/-	-/-	-/-	-/-	-/-
GSRSRT (AMBER99SB)	0.94/0.94	0.89/0.90	0.81/0.74	0.47/0.70	0.66/0.83	0.67/0.66	-/-	-/-	-/-	-/-	-/-
KLVFFAE(CHARMM36)	0.88/0.87	0.90/0.91	0.53/0.41	0.85/0.83	0.41/0.78	0.73/0.83	0.84/0.87	0.85/0.66	0.91/0.56	0.82/0.85	0.80/0.81
AIIGLM (CHARMM36)	0.89/0.89	0.88/0.90	0.87/0.86	0.94/0.94	0.93/0.92	0.92/0.93	0.92/0.93	0.90/0.85	0.88/0.87	0.87/0.86	0.86/0.84
NNQQNY (CHARMM36)	0.91/0.90	0.91/0.90	0.90/0.90	0.89/0.87	0.90/0.94	0.91/0.90	0.85/0.86	-/-	-/-	-/-	-/-
VIQVVY (CHARMM36)	0.92/0.92	0.92/0.93	0.89/0.93	0.92/0.92	0.92/0.92	0.92/0.91	0.88/0.89	0.88/0.82	0.89/0.89	0.83/0.84	-/-
VQIVYK (CHARMM36)	0.94/0.93	0.92/0.91	0.92/0.91	0.86/0.92	0.94/0.91	0.94/0.94	0.82/0.91	0.61/0.64	-/-	-/-	-/-
GSRSRT (CHARMM36)	0.86/0.86	0.89/0.86	0.82/0.82	0.78/0.68	-/-	-/-	-/-	-/-	-/-	-/-	-/-
KLVFFAE (GROMOS96 43A1)	0.76/0.82	0.85/0.76	0.86/0.88	0.81/0.86	0.93/0.94	0.83/0.82	-/-	-/-	0.91/0.91	-/-	0.88/0.87
AIIGLM (GROMOS96 43A1)	0.72/0.64	0.69/0.73	0.62/ 0.63	0.72/0.78	0.74/ 0.75	-/-	-/-	0.86/ 0.83	0.80/0.81	0.83/0.85	0.82/0.83
NNQQNY (GROMOS96 43A1)	0.80/0.82	0.82/0.86	0.87/0.84	0.88/0.83	-/-	0.85/0.84	-/-	0.94/0.94	-/-	-/-	0.92/0.87
VIQVVY (GROMOS96 43A1)	0.71/0.74	0.76/0.67	0.66/0.68	0.88/0.88	0.86/0.82	0.75/0.74	-/-	0.92/0.93	-/-	0.86/0.85	0.83/0.85
VQIVYK (GROMOS96 43A1)	0.80/0.83	0.77/0.80	0.90/0.89	-/-	0.89/0.92	-/-	-/-	0.85/0.86	-/-	-/-	0.88/0.88
GSRSRT (GROMOS96 43A1)	0.70/0.74	0.87/0.81	0.80/0.78	0.88/0.88	0.78/0.82	0.87/0.87	0.81/0.80	0.72/0.73	0.71/0.69	0.69/0.72	-/-

Table 3. Reference structure motifs from crystallographic and NMR data used for RMSD and PCA analysis.

Sequence	PDB ID	Type of motif / No. of conformers
**KLVFFA**	2Y29, 3OW9 (1)	intra-sheet (1); inter-sheet (2)
	2LMN (15)	intra-sheet (1)
	3Q9H (16)	intra-sheet (1); inter-sheet (2)
	4IVH (17)	inter-sheet (1)
**AIIGLM**	2Y3J (1)	intra-sheet (1); inter-sheet (1)
	2LNQ (18)	intra-sheet (1)
	3Q9J (16)	inter-sheet (2)
	3T4G (19)	intra-sheet (1); inter-sheet (1)
**NNQQNY**	1YJO (20)	intra-sheet (1); inter-sheet (1)
	2OMM (2)	inter-sheet (1)
**VQIVYK**	2ON9 (2)	intra-sheet (1); inter-sheet (1)
	3Q9G (16)	intra-sheet (1); inter-sheet (1)
	4E0M (21)	intra-sheet (1); inter-sheet (2)
**FGAILS**	2KIB (22)	intra-sheet (1); inter-sheet (1)
**NFGAIL**	5E5V (23)	intra-sheet (1); inter-sheet (1)
**IAALLS**	2M5K (24)	inter-sheet (1)
**VMVGVV**	2OKZ (2)	inter-sheet (1)
**SNQNNF**	2OL9 (2)	inter-sheet (1)
**VEALYL**	2OMQ (2)	inter-sheet (1)
**GGVVIA**	2ONV (2)	inter-sheet (1)
**SSTSAA**	2ONW (2)	inter-sheet (1)
**NFGAIL**	3DGJ (25)	inter-sheet (1)
**GYVLGS**	3NHD (26)	inter-sheet (1)
**KVLGDV**	3SGO (27)	intra-sheet (2)
**KDWSFY**	4E0K (21)	inter-sheet (2)
**FYLLYY**	4E0L (21)	inter-sheet (2)
**HDCVNI**	4E1H (28)	intra-sheet (1)
**Total no. of conformers/motifs**		**44**

Supplementary Figures

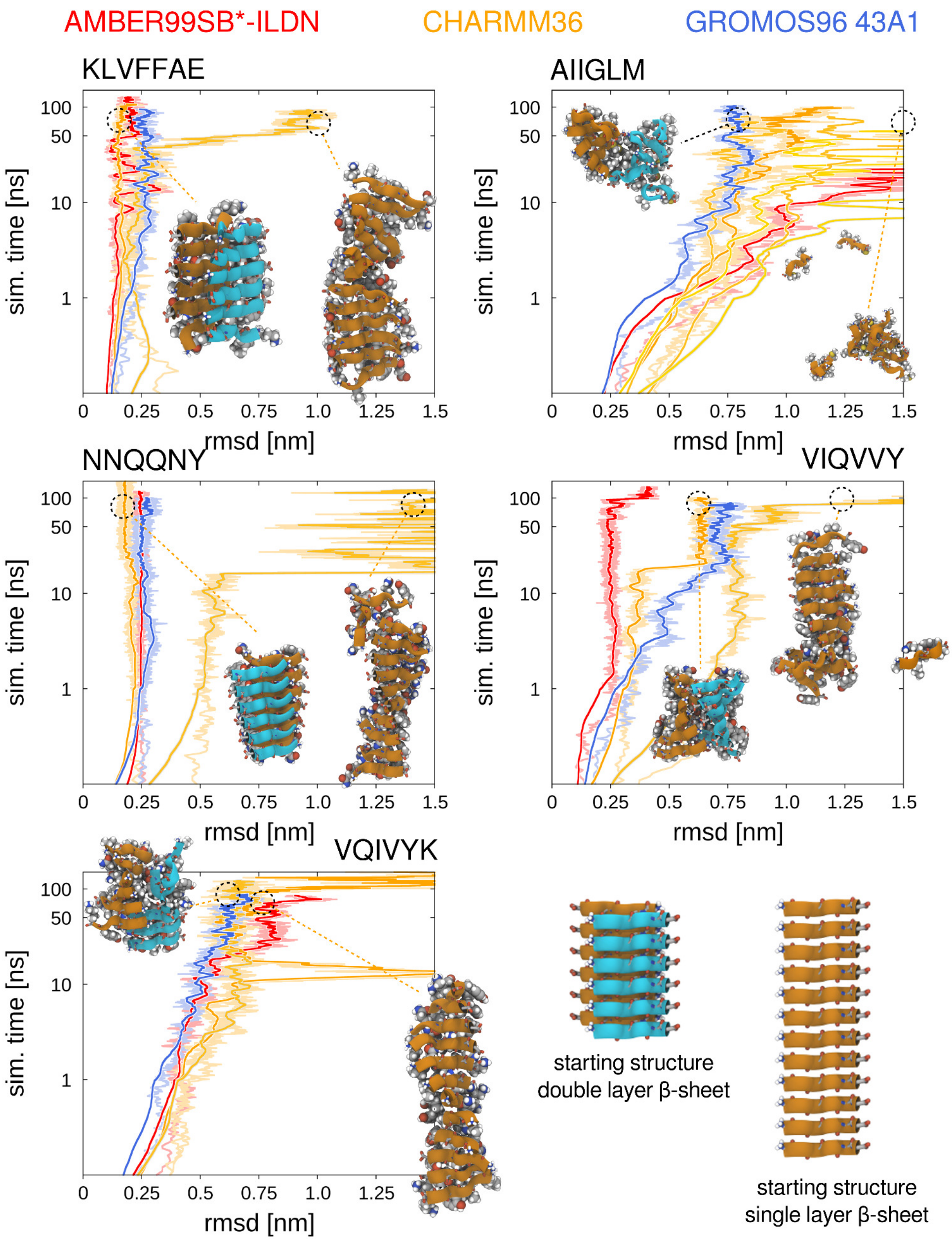

Figure 1. Simulations of single and double layer β-sheet model structures. The RMSD of main-chain and Cβ atoms from the starting structure are shown as function of simulation time. To improve clarity a running average over 5 ns is shown. White outline of the curves denotes simulations of double layer, gray outline simulations of single layer β-sheet oligomers. Red, orange and blue colours denote simulations with AMBER99SB*-ILDN, CHARMM36 and GROMOS96 force fields, respectively.

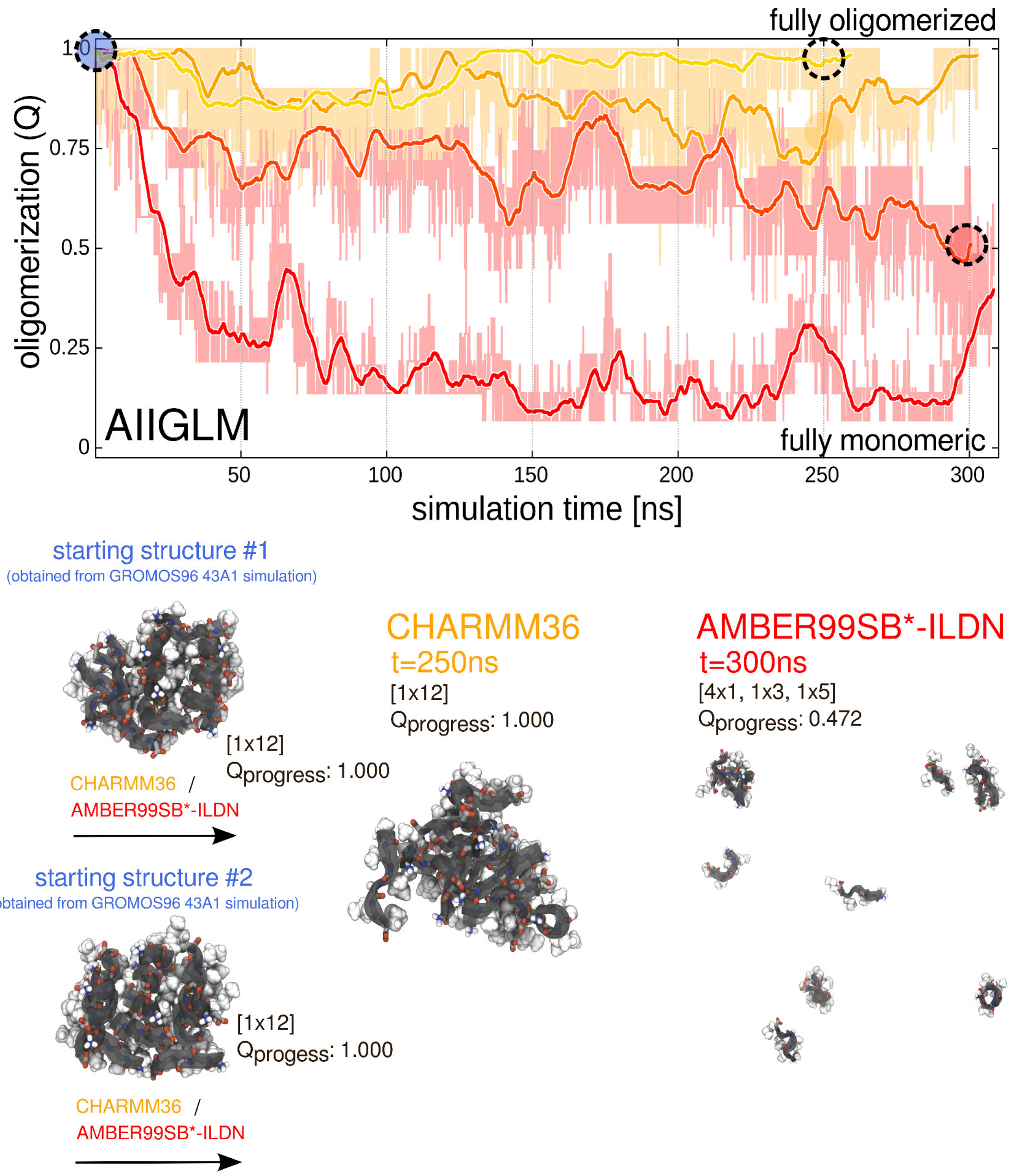

Figure 2. Comparison of AIIGLM peptide association propensity. Association state (Q) for a set of aggregated AIIGLM peptide conformations sampled from GROMOS96 trajectories and simulated in AMBER99SB*-ILDN (red traces) and CHARMM36 force fields (orange traces). A running average over 5 ns is shown to improve clarity.

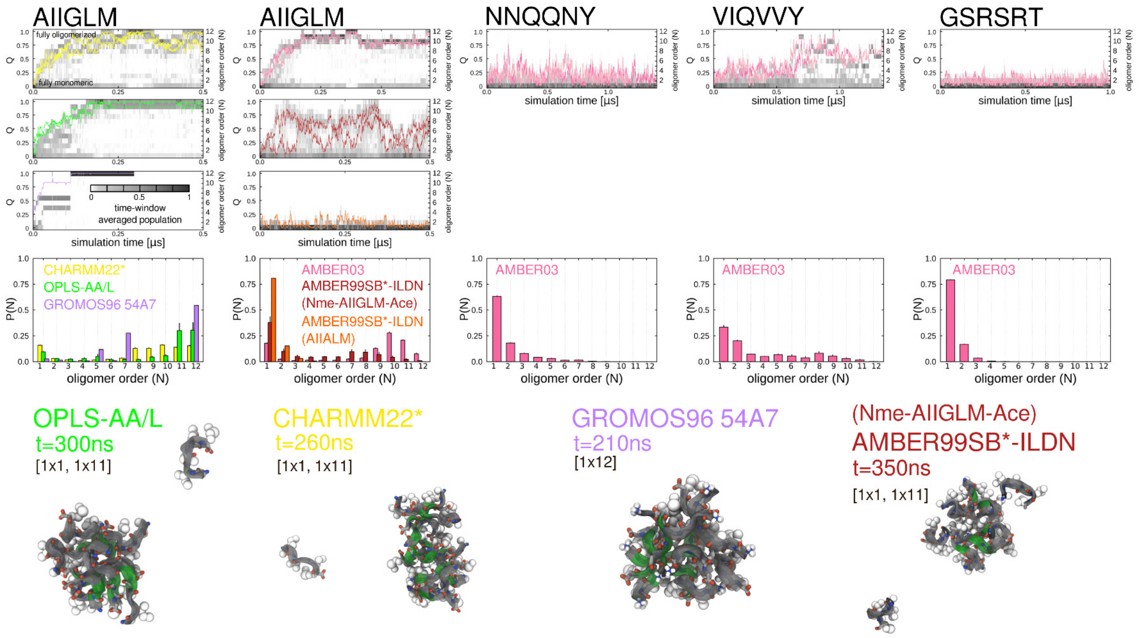

Figure 3. Comparison of oligomerization propensity in various force fields. (A) Oligomerization progress is shown for a set of peptide sequences with the AMBER03, OPLS-AA/L, CHARMM22* and GROMOS96 54A7 force field in addition to simulations in AMBER99SB*-ILDN with blocked termini at 20 mM peptide concentration. Average populations of individual oligomerization states are shown as a function of simulation time averaged over all trajectories. Dark-gray and gray colours indicate a high abundance of oligomer order N. Respective block averages over 10 ns are shown to improve clarity. (B) Bar histograms in each multi panel depict the normalized oligomer size distributions determined over all trajectories of a particular sequence and force field combination.

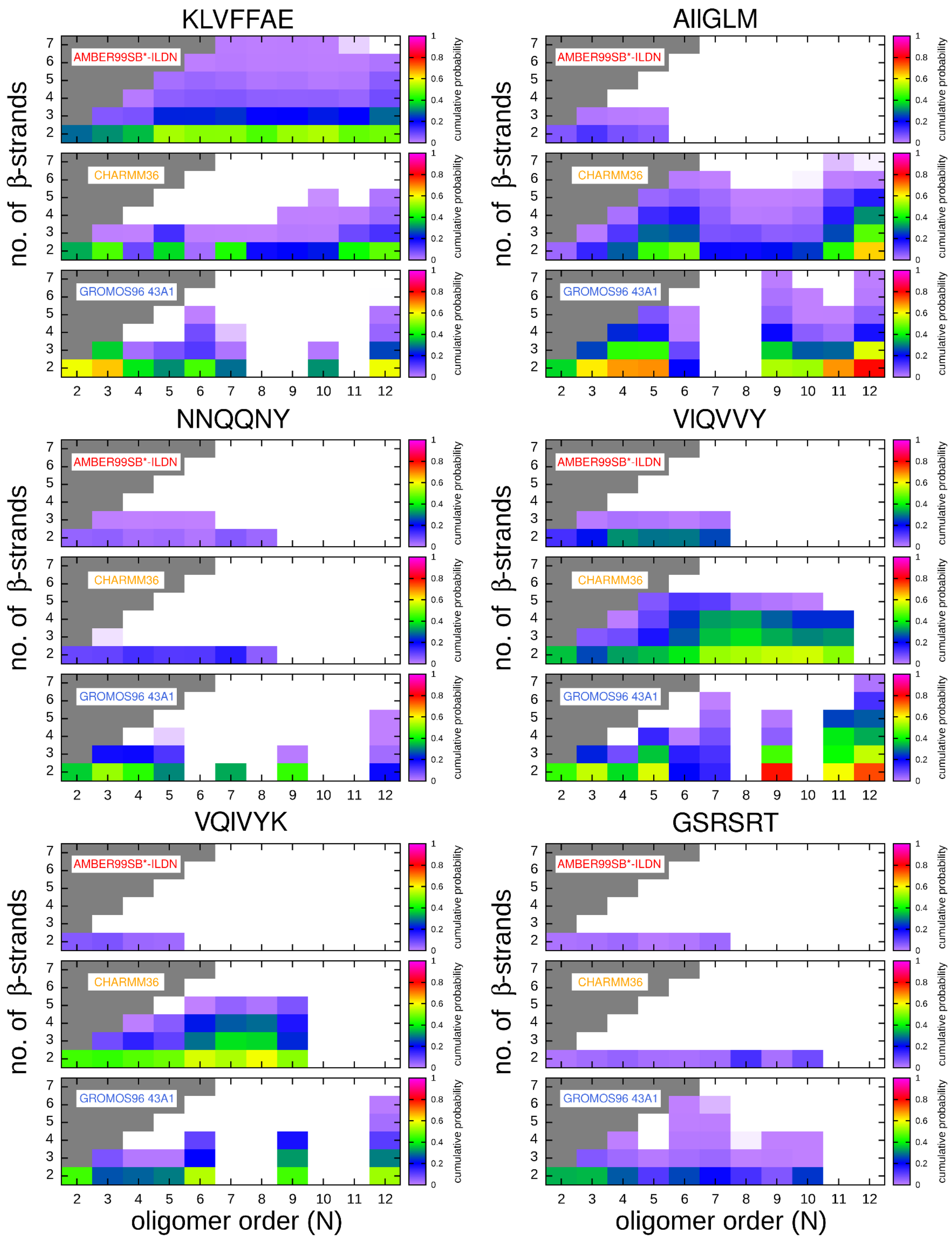

Figure 4. β-sheet subunit size per aggregate size. Histograms of β-sheet subunit size within each oligomeric state of order N for simulations carried out with AMBER99SB*-ILDN, CHARMM36 and GROMOS96 force fields. The cumulative probability of all β-sheet subunit sizes present for a particular oligomer size N are always summed starting from the largest to the smallest subunit with a non-zero probability. β-sheet subunit sizes that were not sampled are coloured in white.

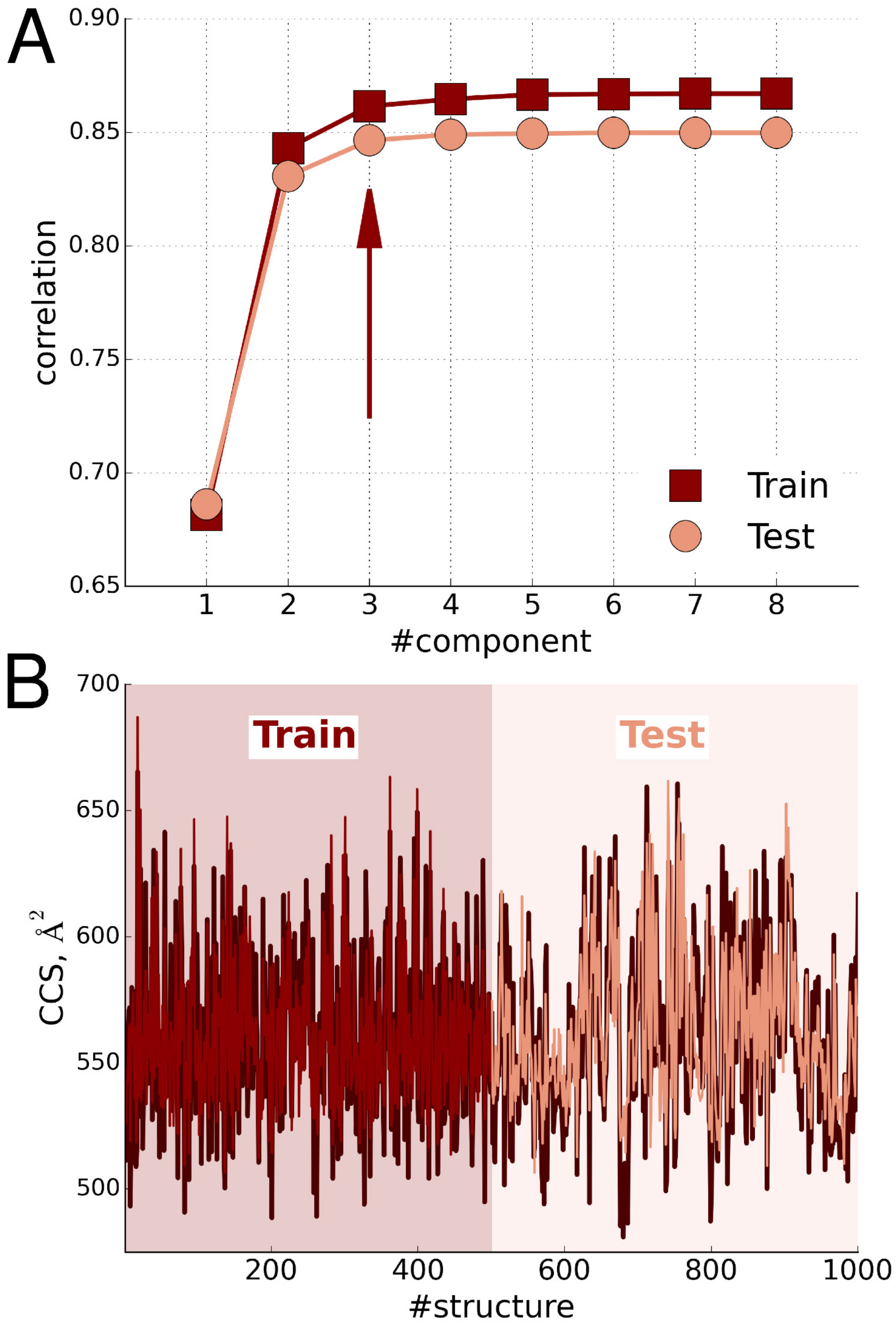

Figure 5. Example of Partial Least Square (PLS) model building for the prediction of collision cross sections (CCSs). (A) Change of the correlation coefficients between the calculated and predicted CCS values to determine the optimal number of PLS components. (B) PLS models were built on one half of the available CCSs calculated by the Trajectory method, the other half of the CCS data was used for testing and cross-validation.

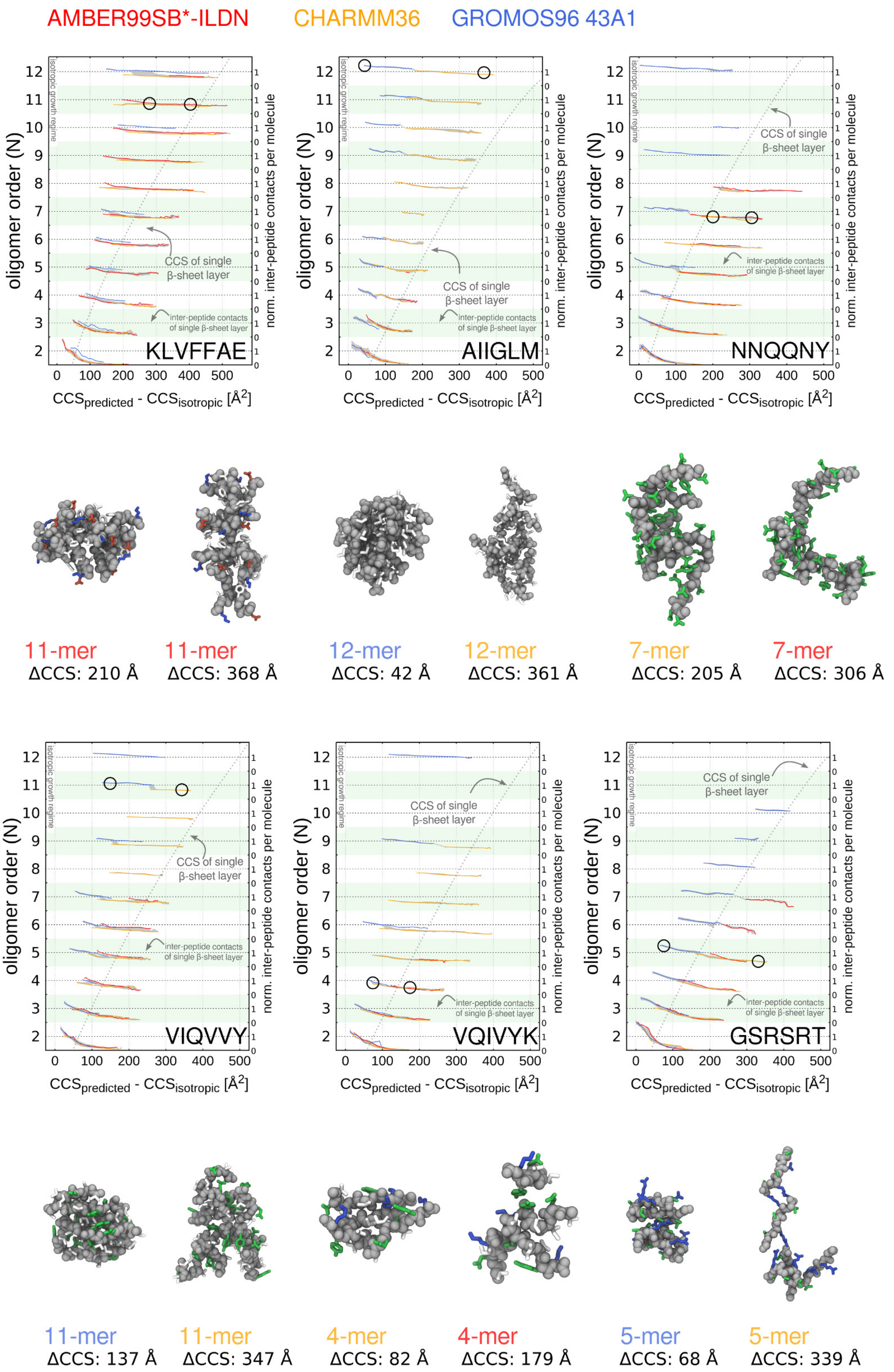

Figure 6. Inter-peptide contact analysis. Top panels show the averaged inter-peptide heavy atom contacts to illustrate the packing density for the oligomeric aggregates of each state. Red, orange and blue coloured lines denote the distribution medians obtained from AMBER99SB*-ILDN, CHARMM36 and GROMOS96 simulations. The gray area shows the interquartile range over the distributions of all simulations. Selected structure representatives are shown in spheres for main-chain atoms and as sticks for side-chain atoms. The side-chains atoms are coloured according to their polarity (blue - positively charged, red - negatively charged, green - hydrophilic, white - hydrophobic).

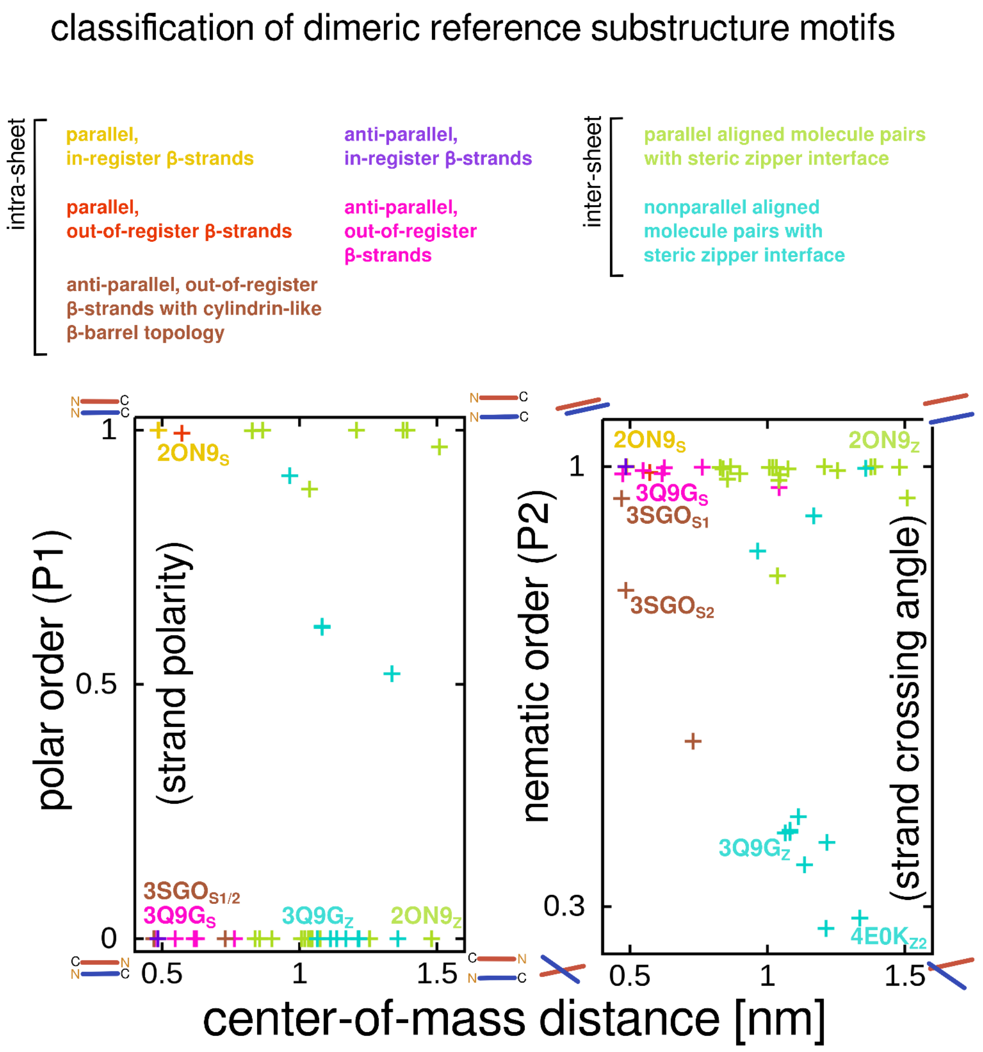

Figure 7. Reference structure motifs from crystallographic and NMR data.

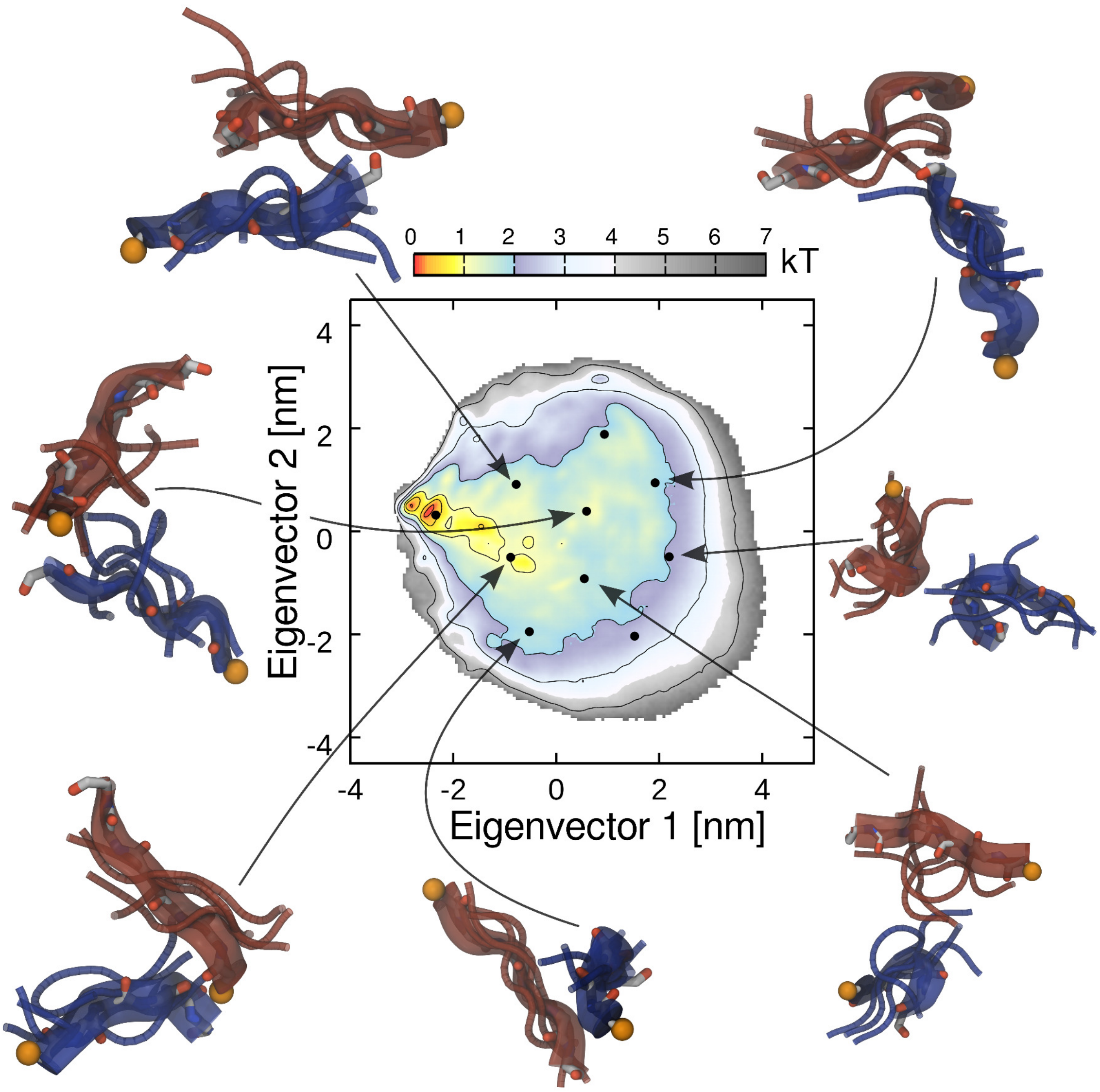

Figure 8. Representative structures of oligomer substructures obtained by conformational clustering. PCA projection of canonical dimeric substructure conformations is shown as density plots (data pooled over all simulations oligomer sizes, sequences and force fields). Black filled circles denote location of cluster centers obtained by k-means clustering. Structure representatives for conformational clusters are shown as ribbons with main-chain atoms shown as sticks. The N-terminal nitrogen atoms of each chain are highlighted by orange spheres.

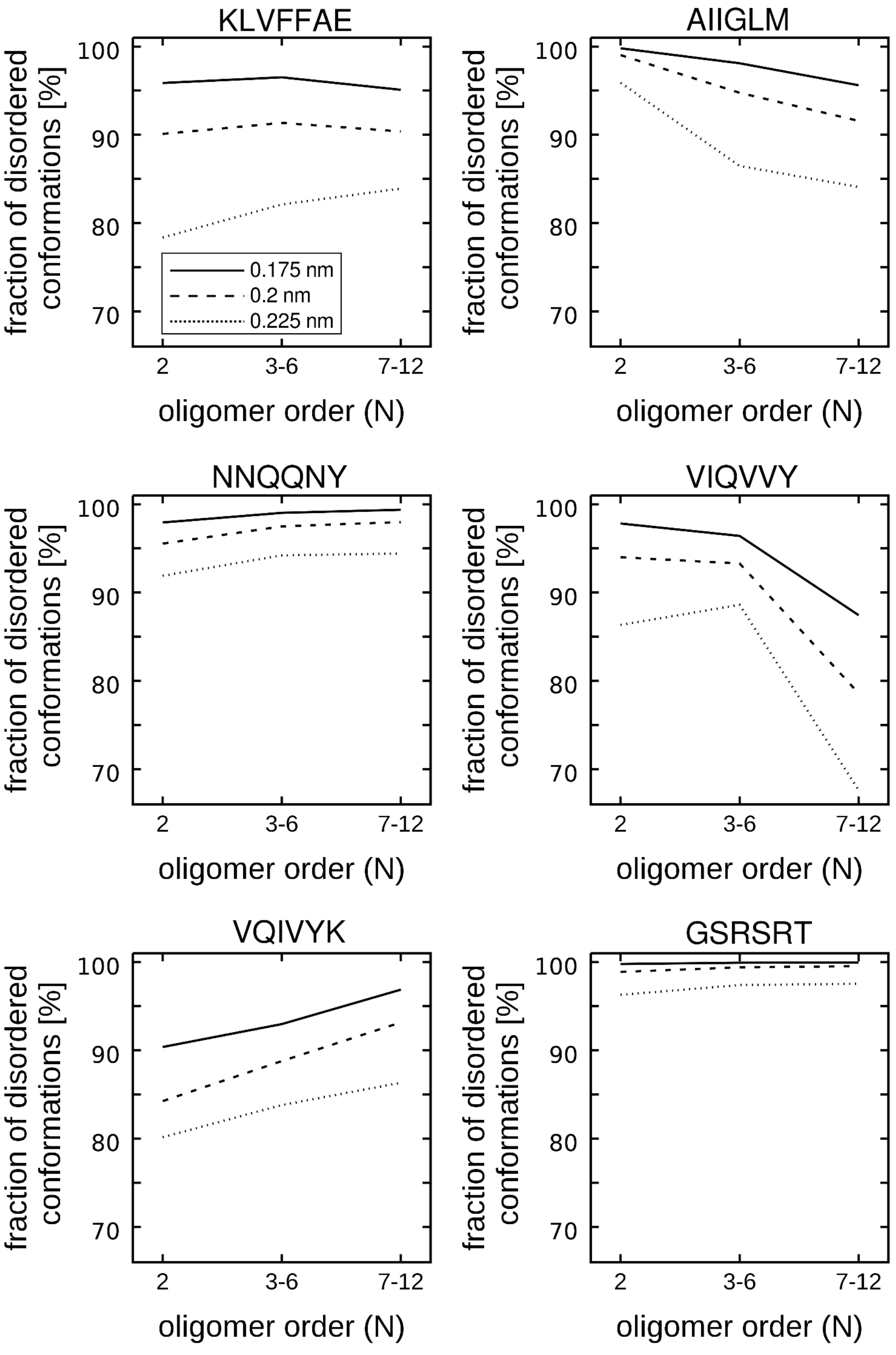

Figure 9. Fraction of disordered local substructures. Fraction of disordered local substructures in the ensemble of sampled substructure conformations that could not be assigned to any of the seven reference motifs using three different RMSD cut-offs.

References

1. Colletier, J.-P. et al. Molecular basis for amyloid-beta polymorphism. Proc. Natl. Acad. Sci. USA **41**, 16938–16943 (2011).

2. Sawaya, M. R. et al. Atomic structures of amyloid cross-beta spines reveal varied steric zippers. Nature **447**, 453–457 (2007).

3. Goldschmidt, L., Teng, P. K., Riek, R. & Eisenberg, D. Identifying the amylome, proteins capable of forming amyloid-like fibrils. Proc. Natl. Acad. Sci. USA **107**, 3487–3492 (2010).

4. Bussi, G., Donadio, D. & Parrinello, M. Canonical sampling through velocity rescaling. J. Chem. Phys. **126**, 014101 (2007).

5. Hess, B. P-lincs: a parallel linear constraint solver for molecular simulation. J. Chem. Theor. Comput. **4**, 116–122 (2008).

6. Miyamoto, S. & Kollman, P. A. Settle: An analytical version of the shake and rattle algorithm for rigid water models. J. Comput. Chem. **13**, 952–962 (1992).

7. Darden, T., York, D. & Pedersen, L. Particle mesh ewald: An n-log(n) method for ewald sums in large systems. J. Chem. Phys. **98**, 10089–10092 (1993).

8. Essmann, U. et al. A smooth particle mesh Ewald method. J. Chem. Phys. **103**, 8577–8593 (1995).

9. Feenstra, K. A., Hess, B. & Berendsen, H. J. C. Improving efficiency of large time-scale molecular dynamics simulations of hydrogen-rich systems. J. Comput. Chem. **20**, 786–798 (1999).

10. Parrinello, M. & Rahman, A. Polymorphic transitions in single crystals: A new molecular dynamics method. J. Appl. Phys. **52**, 7182 (1981).

11. Pronk, S. et al. Gromacs 4.5: a high-throughput and highly parallel open source molecular simulation toolkit. Bioinformatics **29**, 845–854 (2013).

12. Berendsen, H. J. C., Postma, J. P. M., van Gunsteren, W. F., DiNola, A. & Haak, J. R. Molecular dynamics with coupling to an external bath. J. Chem. Phys. **81**, 3684–3690 (1984).

13. Eisenberg, D. & McLachlan, A. D. Solvation energy in protein folding and binding. Nature **319**, 199–203 (1986).

14. Espinosa, E., Molins, E. & Lecomte, C. Hydrogen bond strengths revealed by topological analyses of experimentally observed electron densities. Chem. Phys. Lett. **285**, 170–173 (1998).

15. Paravastu, A. K., Leapman, R. D., Yau, W.-M. & Tycko, R. Molecular structural basis for polymorphism in alzheimer's beta-amyloid fibrils. Proc. Natl. Acad. Sci. USA **105**, 18349–18354 (2008).

16. Liu, C. et al. Characteristics of amyloid-related oligomers revealed by crystal structures of macrocyclic beta-sheet mimics. J. Am. Chem. Soc. **133**, 6736–6744 (2011).

17. Pham, J. D., Chim, N., Goulding, C. W. & Nowick, J. S. Structures of oligomers of a peptide from beta-amyloid. J. Am. Chem. Soc. **135**, 12460–12467 (2013).

18. Qiang, W., Yau, W.-M., Luo, Y., Mattson, M. P. & Tycko, R. Antiparallel beta-sheet architecture in iowa-mutant beta-amyloid fibrils. Proc. Natl. Acad. Sci. USA **109**, 4443–4448 (2012).

19. Cheng, P.-N., Liu, C., Zhao, M., Eisenberg, D. & Nowick, J. S. Amyloid beta-sheet mimics that antagonize protein aggregation and reduce amyloid toxicity. Nat. Chem. **4**, 927–933 (2012).

20. Nelson, R. et al. Structure of the cross-beta spine of amyloid-like fibrils. Nature **435**, 773–778 (2005).

21. Liu, C. et al. Out-of-register beta-sheets suggest a pathway to toxic amyloid aggregates. Proc. Natl. Acad. Sci. USA **109**, 20913–20918 (2012).

22. Nielsen, J. et al. Unique identification of supramolecular structures in amyloid fibrils by solid-state nmr spectroscopy. Angew. Chem., Int. Ed., **48**, 2118–2121 (2009).

23. Soriaga, A. B., Sangwan, S., Macdonald, R., Sawaya, M. R. & Eisenberg, D. Crystal structures of iapp amyloidogenic segments reveal a novel packing motif of out-of-register beta sheets. J. Phys. Chem. B **Article ASAP** (2016).

24. Fitzpatrick, A. W. P. et al. Atomic structure and hierarchical assembly of a cross-beta amyloid fibril. Proc. Natl. Acad. Sci. USA **14**, 5468–5473 (2013).

25. Wiltzius, J. J. W. et al. Molecular mechanisms for protein-encoded inheritance. Nat. Struct. Mol. Biol. **16**, 973–978 (2009).

26. Apostol, M. I., Sawaya, M. R., Cascio, D. & Eisenberg, D. Crystallographic studies of prion protein (prp) segments suggest how structural changes encoded by polymorphism at residue 129 modulate susceptibility to human prion disease. J. Biol. Chem. **285**, 29671–29675 (2010).

27. Laganowsky, A. et al. Atomic view of a toxic amyloid small oligomer. Science **335**, 1228–1231 (2012).

28. Apostol, M. I., Perry, K. & Surewicz, W. K. Crystal structure of a human prion protein fragment reveals a motif for oligomer formation. Journal of the American Chemical Society **135**, 10202–10205 (2013).
